# Agent-based models of malaria transmission: a systematic review

**DOI:** 10.1186/s12936-018-2442-y

**Published:** 2018-08-17

**Authors:** Neal R. Smith, James M. Trauer, Manoj Gambhir, Jack S. Richards, Richard J. Maude, Jonathan M. Keith, Jennifer A. Flegg

**Affiliations:** 1 School of Public Health and Preventive Medicine, Monash University, Melbourne, Australia; 2 IBM Research Australia, Melbourne, Australia; 3 Life Sciences, Burnet Institute, Melbourne, Australia; 4 Department of Medicine, University of Melbourne, Parkville, Australia; 5 Department of Infectious Diseases, Monash University, Melbourne, Australia; 6 Mahidol-Oxford Tropical Medicine Research Unit, Faculty of Tropical Medicine, Mahidol University, Bangkok, Thailand; 7 Centre for Tropical Medicine and Global Health, Nuffield Department of Medicine, University of Oxford, Oxford, UK; 8 Harvard TH Chan School of Public Health, Harvard University, Boston, USA; 9 School of Mathematical Sciences, Monash University, Clayton, Australia; 10 School of Mathematics and Statistics, University of Melbourne, Parkville, Australia

**Keywords:** Malaria, Infectious disease transmission, Agent-based model, Individual-based model, Review

## Abstract

**Background:**

Much of the extensive research regarding transmission of malaria is underpinned by mathematical modelling. Compartmental models, which focus on interactions and transitions between population strata, have been a mainstay of such modelling for more than a century. However, modellers are increasingly adopting agent-based approaches, which model hosts, vectors and/or their interactions on an individual level. One reason for the increasing popularity of such models is their potential to provide enhanced realism by allowing system-level behaviours to emerge as a consequence of accumulated individual-level interactions, as occurs in real populations.

**Methods:**

A systematic review of 90 articles published between 1998 and May 2018 was performed, characterizing agent-based models (ABMs) relevant to malaria transmission. The review provides an overview of approaches used to date, determines the advantages of these approaches, and proposes ideas for progressing the field.

**Results:**

The rationale for ABM use over other modelling approaches centres around three points: the need to accurately represent increased stochasticity in low-transmission settings; the benefits of high-resolution spatial simulations; and heterogeneities in drug and vaccine efficacies due to individual patient characteristics. The success of these approaches provides avenues for further exploration of agent-based techniques for modelling malaria transmission. Potential extensions include varying elimination strategies across spatial landscapes, extending the size of spatial models, incorporating human movement dynamics, and developing increasingly comprehensive parameter estimation and optimization techniques.

**Conclusion:**

Collectively, the literature covers an extensive array of topics, including the full spectrum of transmission and intervention regimes. Bringing these elements together under a common framework may enhance knowledge of, and guide policies towards, malaria elimination. However, because of the diversity of available models, endorsing a standardized approach to ABM implementation may not be possible. Instead it is recommended that model frameworks be contextually appropriate and sufficiently described. One key recommendation is to develop enhanced parameter estimation and optimization techniques. Extensions of current techniques will provide the robust results required to enhance current elimination efforts.

**Electronic supplementary material:**

The online version of this article (10.1186/s12936-018-2442-y) contains supplementary material, which is available to authorized users.

## Background

Malaria, alongside HIV and tuberculosis, is considered one of the “big three” infectious diseases of humans. The global response to malaria transmission has been significant, with *Plasmodium falciparum* malaria eliminated from 79 countries from 1979 to 2010 [1]. Modelling suggests that 70% of the reduction in malaria cases in sub-Saharan Africa (SSA) between 2000 and 2015 was attributable to the implementation of intervention strategies [2]. Key interventions included insecticide-treated bed nets (ITNs), artemisinin-based combination therapy (ACT) and indoor residual spraying (IRS). Often, field data are used as evidence for the efficacy and cost-effectiveness of selected interventions; however, these methods can be resource intensive, or have prohibitive ethical barriers. In such situations, mathematical simulation is increasingly used to provide further insights.

Infectious disease modelling of malaria has existed for over a century [3], with the dominant paradigm being the Ross–Macdonald models used by the Global Malaria Eradication Programme (GMEP) from 1955 to 1969 [4, 5]. These are examples of compartmental transmission dynamic models, in which the simulated human population consists of groups of individuals in disease states such as “susceptible”, “exposed”, “infectious” and “recovered”. More recent compartmental models of malaria provide insights into risk-stratification of populations, multiple mosquito populations, and waning immunity; however, the majority of models still closely resemble the Ross–Macdonald configuration [6]. Comparing a variety of modelling approaches can provide robustness of results, and highlight areas for development of modelling techniques [7]. As such, comparing alternative model frameworks may accelerate learning about disease transmission and control.

One approach that has significant potential is the use of agent-based models (ABMs). There appears to be no universal definition of ABMs; this review includes any model that explicitly models individual actions and responses, and associates with each individual respective state variables and parameters. In the following review, all published ABMs are stochastic in nature.

As ABMs focus on the individual, they afford flexibility in modelling factors, such as spatial heterogeneity (e.g. host movement, heterogeneous implementation of interventions) and stochasticity (e.g. inter-patient variability in time of infection, time to recovery, and location of infection). Compartmental models of malaria transmission do exist that incorporate either stochasticity of individual infections [8] or spatial heterogeneity [9]. However, in areas of increasing spatial variation, compartmental models may face convergence issues, or provide no more insight than alternative model structures. In low-transmission environments, where patient variability is more pronounced, ABMs can better represent the stochasticity of disease progression and transmission than compartmental models, where (to some resolution) people are grouped together and treated as interchangeable, so individual agent behaviour cannot be determined. Models that accommodate patient individuality and spatial variation can help fill knowledge gaps [7] about transmission heterogeneities important in malaria elimination strategies.

The flexibility of agent-based approaches also allows models to be constructed to address practical questions relating to malaria control and elimination in specific local contexts [7, 10]. This is advantageous because identifying optimal local intervention strategies can provide a strong evidence-based framework for National Malaria Control Programmes (NMCPs). ABMs can be constructed to resemble such specific settings closely, due to their flexibility in altering model attributes to reflect local individual characteristics and geographical factors.

As more is learned about malaria transmission, the complexity of the questions asked increases, which in turn calls for more nuanced models. The role of mathematical models continues to grow as both technical expertise and computing power increase. With the increasing capacity for modelling to assist in malaria elimination programmes, a review of the published literature for ABMs of malaria transmission was performed. Analysis included characterization of the structure of existing models, the factors influencing malaria transmission modelled, and the methods of data use and output analysis. The approaches used were highlighted and ideas for advancing the field proposed.

## Methods

### Search strategy and selection criteria

A systematic literature review was performed, consistent with the PRISMA statement [11]. Database searches of OVID Medline, CINAHL Plus and OVID Embase were performed as outlined in Additional file 1.

The search strategy aimed to return publications referring to each of the following three concepts in their subject heading, keywords list, title or abstract:

1. Terms relating to malaria, malaria vaccines, anti-malarials (including individual medications), and relevant *Plasmodium* species
2. Terms relating to epidemiology, demography, infectious disease outbreaks or transmission, epidemics and key outbreak model parameters (e.g. basic reproduction number)
3. Terms relating to agent-based, individual-based or microsimulation models.

No limits were placed on publication type, language, location, dates or publication status. Further to database searching, additional articles were found through expert knowledge, and backward and forward citation searching (the latter using Google Scholar) of included articles.

The abstracts of all returned studies were assessed for suitability, with those that mentioned an agent-based, individual-based, or microsimulation model of malaria transmission selected for full-text review. Full-text articles were excluded if no agent-based model of hosts or vectors was described or used, or if no components of malaria transmission (such as human disease, vector biting, or interventions) were explicitly modelled. Models of vector life cycles or ecology without malaria-specific elements were excluded. Articles that described comparison or ensemble modelling of pre-existing models were considered separately, and were not “included studies” for the purposes of this review.

### Data collection and analysis

Due to the anticipated variation within the literature, a data extraction form was not developed prospectively. Instead, for included publications, key characteristics of each model were identified, with common themes and unique properties of models characterized. After model themes were identified, a classification for key components of models was created (see Additional file 2). The properties of each model were collated as discussed in the “Results” section.

## Results

The search yielded 406 abstracts potentially meeting inclusion criteria; 137 were selected for full-text assessment, with 90 papers included in the final analysis. Search results are presented diagrammatically using the PRISMA template (available at: http://www.prisma-statement.org/) in Additional file 3. The table in Additional file 2 provides detailed information about each model.

## Model construction

### Development of models and model families

**Fig. 1:**
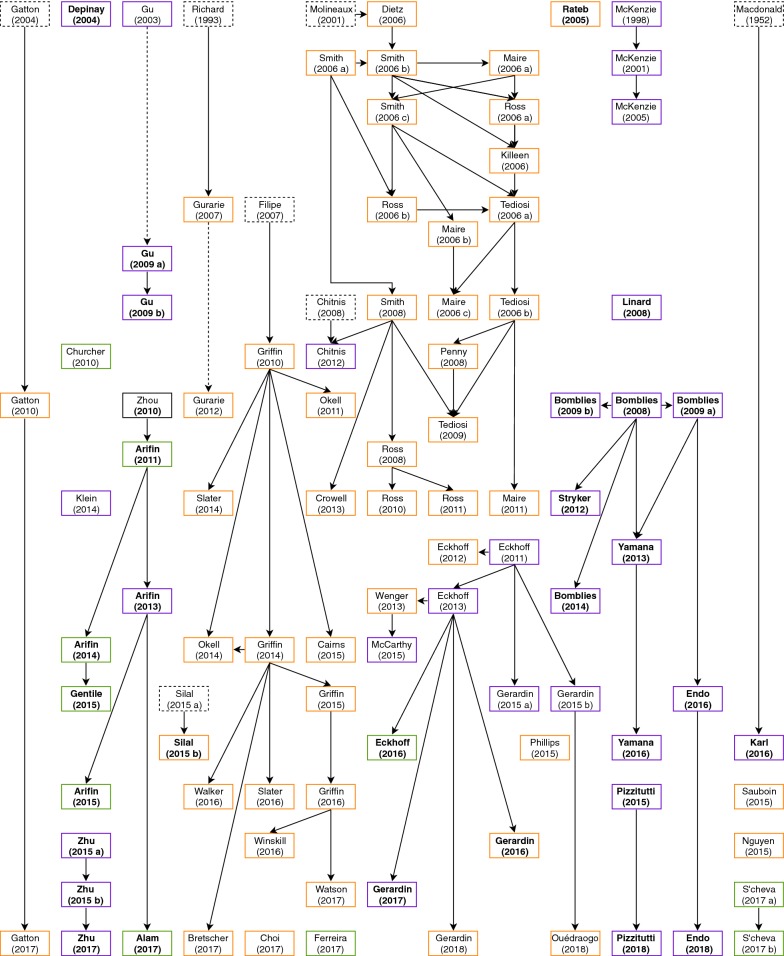
Overview of included studies. The most recent studies are towards the bottom of the diagram. Solid arrows indicate papers directly linked to one another. Dotted arrows indicate models by the same lead author that are not related in methodology. Black solid or dotted boxes indicate models that did not meet inclusion criteria or are not agent-based, respectively, that guided the creation of a later ABM. Papers named in bold are spatially explicit. Boxes coloured orange, green and purple include human agents, mosquito agents or both, respectively

Figure 1 outlines the timeline of models in the included papers. 27 original model frameworks have been published, with 15 models reported once to date, and 12 ‘baseline’ models collectively resulting in 63 further publications. Of the 27 original ABMs, six models were developed by extending compartmental or within-host models to an individualized framework [12–17]. Model progression was largely similar across the largest model frameworks, including OpenMalaria [18], EMOD [19] HYDREMATS [20], and work from Imperial College [14], such that the topic of each paper progressed logically from previous works. Extensions included simulating interventions [21], spatial mapping [22], or embedding “sub-modules” of key disease concepts into pre-existing works, such as host infectivity [23], mortality [24], and potential impacts of climate change [25]. When interventions were adequately simulated, cost-effectiveness analysis (CEA) was sometimes performed [26–28].

### Model structure and frameworks

**Fig. 2:**
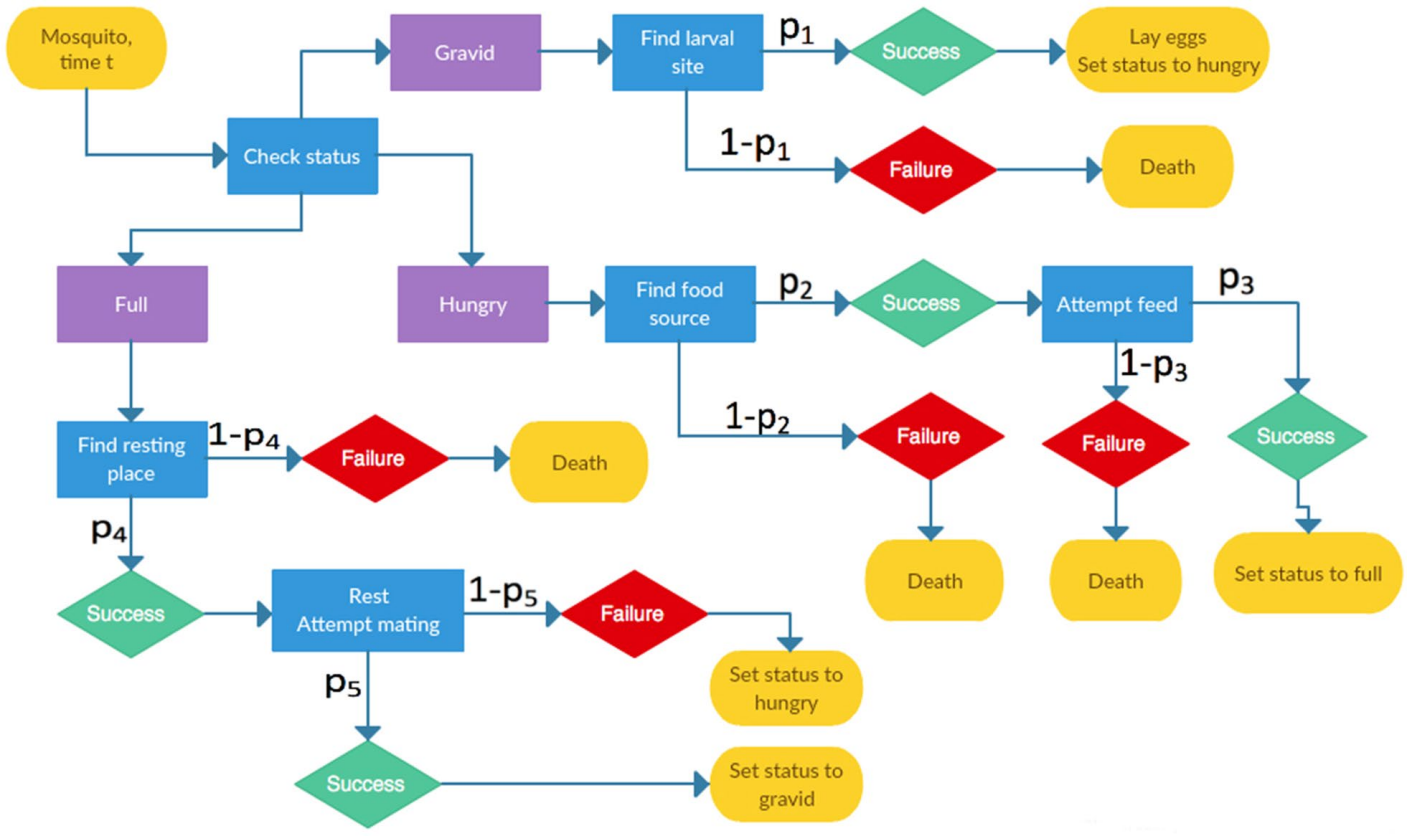
A hypothetical ‘decision tree’ approach to modelling mosquito agents. At each time step t, mosquitoes will check their status, determining their subsequent action(s) *i* with probability p_i_ of success. Individuals may have identical or differing probabilities of success for each task

Methods of modelling at the individual level are influenced by factors including the question at hand, characteristics of the agent, and interactions of interest. Certain ABM frameworks naturally arise from the above concepts; in malaria modelling, key considerations include the choice of agent and whether to focus on disease states or transmission. While these choices are not mutually exclusive, three broad methods of individual simulation were common in the literature, each with differing degrees of agent autonomy. First, models developed from a compartmental structure typically used probabilities in place of flow rates to determine whether an individual transitioned to a new disease state at a given time step (e.g. Gurarie and McKenzie [13], and McKenzie et al. [29]). Using this method, the success or failure of a Bernoulli trial generally dictated disease progression. Second, and particularly in models focused on host parasite densities (see the OpenMalaria simulations [18]), distributions of key variables were sampled to generate differences between agents, with temporal disease state changes governed by a set of equations. In the third method, the specific actions of individuals, for example blood meal searching, resting and oviposition, were simulated according to a process represented by a flow chart (e.g. Pizzitutti et al. [30], Zhu et al. [31] or the EMOD framework [19]). Figure 2 presents a hypothetical flow chart of vector actions, to illustrate a typical set of transitions available to a mosquito.

The diversity of methods led to a broad range of agent constructions, affecting the number and type of variables, the variation between individuals, and the type of agents required. Because methods to construct ABMs are so broad, two examples are provided below for comparison. These two models are examples of ABMs being tailored to the question at hand and highlights the variation in model construction.

Consider an early OpenMalaria simulation of the relationship between the entomological inoculation rate (EIR) and force of infection [18]. Human agents had individual ages, leading to an age-adjusted EIR. Individual mosquitoes were not explicitly modelled; instead, EIR was used to guide biting rates [32]. Two individuals of the same age may have a different number of infections on a particular day, but these numbers are drawn from the same distribution [33]. As OpenMalaria grew to answer new questions, these individuals also had different gametocyte densities [34], pyrogenic thresholds [35], and morbidity and mortality [24].

In contrast, Zhu et al. [21, 31, 36] developed a spatial ABM to investigate the impact of the location of food, hosts, and resting and breeding sites on malaria transmission. This required simulation of a physical landscape, with both mosquito and human locations guided by movement patterns. Mosquito agents had a sequence of actions to be undertaken, and therefore required variables capturing their current hunger, breeding state, and flight distance since last meal. The number of bites for each human was also recorded. The distance to the target of interest (sugar, host, resting site, breeding site) and time of day could dictate vector flight paths. Capping the number of eggs per larval habitat required the current number of eggs to be recorded.

There were also considerations relating the method of agent simulation and size of the time step. Where compartmental models inspired an ABM, daily time steps were most common [15]. To simulate host infections using distributions for variables, a temporal resolution of 1–5 days was sufficient to represent changing disease characteristics [32]. However, if agent actions were explicitly modelled, hourly status updates were most common, with some models tracking vectors as frequently as each second [21, 31].

## Agency and elements modelled

**Fig. 3:**
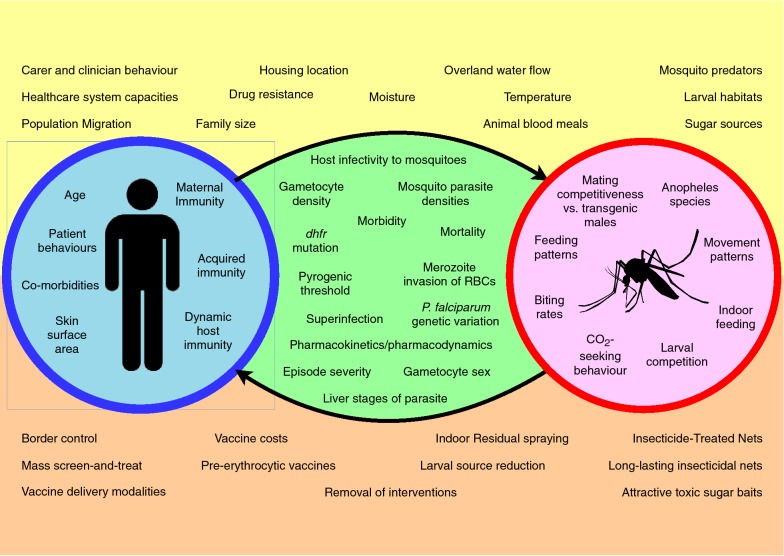
Diagram outlining factors influencing malaria transmission that have been modelled by ABMs. Factors pertaining to humans and mosquitoes are in red and blue circles, respectively. Factors about the disease process are within the arrows linking these circles. Factors at the top and bottom of the diagram are environmental factors and interventions, respectively

The complexity of malaria transmission prevents any one model simulating all transmission factors in depth. In practice, each framework is centred on a few core concepts; these are generally sources of heterogeneity that motivate ABM use. These concepts can be categorized as *host*, *vector*, *parasite*, *environment*, and *intervention* factors. The core components of a model appeared to dictate the characteristics of each model framework and this relationship is discussed in the following sections. Figure 3 outlines some of the factors that vary between transmission scenarios, and therefore may be suited to modelling with an ABM.

Model agents were either humans only (49/90 models), mosquitoes only (9/90) or both humans and mosquitoes (32/90) (Additional file 2, column 4). In a number of instances, the agency of vectors was unclear, although had been used in precursor studies. Pizzitutti et al. considered a *Plasmodium* agent as the infection of a host or vector [30, 37], whereas other models considered these elements as part of a vector or human agent. The AGiLESim framework specified larval habitats as agents, having properties such as location, larval capacity and current egg population [22]. The HYDREMATS simulations of the Bomblies et al. hydrology model [20, 25, 38–42] describe individual larval habitats with many variables and time-dependent formulae, but did not refer to habitats as agents.

### Host

Models with only humans as agents commonly focused on the impact of infection characteristics or medical interventions on transmission. For example, the OpenMalaria models from the Swiss Tropical and Public Health Institute had at their core individual humans with varying parasite densities [18]. These models were extended to incorporate individual effects of innate immunity [34], pyrogenic thresholds [35] and the impact of vaccination [43], particularly pre-erythrocytic vaccines [18, 33, 44]. The models from this group accounted for 20 of the 49 human-only studies.

Key simulated human factors included age and host immunity, with immunity either being maternal, [13, 14] acquired [18, 39, 45] or both [34]. Details about immunity varied from general descriptions of the impact of immunity on transmission, to individualized antibody concentrations for sexual-stage parasites [46]. Human behaviours regarding intervention use [30] and treatment decisions have been simulated for both patients [47] and carers [28]. One model simulated the impact of anti-malarial use on HIV-positive pregnant women [48], including disease severity and improvements in birth weight.

The most commonly simulated disease aspects were gametocyte density (incorporated in all OpenMalaria models [18]) and the infectiousness of hosts to mosquitoes [49], with other common elements including fever [35], disease morbidity and mortality [24] and disease severity [24, 50].

Of 81 papers with human agents, 50 stratified individuals by age (Additional file 2, column 13), either to vary disease profiles or report results. Commonly, age was used in the calculation of human biting rates, because of its strong correlation with body surface area [18]. Other age-varying factors were adaptive and maternal immunity and duration of infection [17]. If interventions were age-specific, for example vaccination programmes [33], stratification was used to assess the impact at different levels of the community [44].

### Vector

Models with mosquitoes as agents most commonly assessed interventions impacting vector mortality, such as habitat removal [30], IRS [16] or ITN [51]. Detailed models of vector life cycle and ecology were common, with mosquito behaviours, such as feeding habits [31, 52], movement patterns [30, 53] and biting frequency [54], modelled in detail. Vector movement was often modelled as random motion, but CO_2_ gradients were also considered [20, 55–57]. Simulations such as the EMOD framework modelled the egg population as a cohort for their progression to adulthood [19], and two models simulated eggs as individual agents [58, 59].

In most publications, only female mosquitoes were simulated [60, 61], as the sex responsible for transmission. Male mosquitoes were generally modelled when a complete population was required, for example to assess vector control interventions (e.g. [21]). Males were specifically required to examine the effects of introducing gene drive mosquitoes into an environment, such as “driving-Y” modified male vectors [62]. Although dominated by *An. gambiae* representation, models also varied by *Anopheles* species, including *An. vagus* [63], *An. stephensi* [64], *An. arabiensis* [62], and *An. darlingi* [30, 37]. One *An. gambiae* model [65] was adapted to *An. vagus* vectors [63] to simulate transmission in Bangladesh, and several models incorporated multiple *Anopheles* populations [14, 56, 66–68], were adaptable across species [51, 58, 59, 69], or were nonspecific in *Anopheles* species [60, 70, 71].

When both hosts and vectors were simulated, models focused on mosquito life cycles [19], population dynamics [58], physical environments [55] and interventions for vector control [21]. Human agents were often included to relate *Anopheles* populations and malaria transmission, in models with vector dynamics as a core component. When models also included a spatial component, malaria transmission generally required vector and host to be co-located. These simulations used a ‘decision tree’ to represent the timing of movements and other necessary actions [21, 30, 31] (e.g. Fig. 2). Simulation of both hosts and vectors at the individual level was regularly used to assess interventions directed at mosquitoes and their effects on malaria transmission [19, 72].

### Parasite

Of 54 models that specified a malaria parasite (see Additional file 2, column five), 51 modelled the dynamics of *Plasmodium falciparum,* two simulated both *P. falciparum* and *Plasmodium vivax* [30, 37], and one *Plasmodium berghei* [64]. The in-host modelling of *P. falciparum* dynamics by Molineaux et al. [12, 18, 73] was used in 21 papers. In some studies, *Plasmodium* infections within an individual were modelled as agents [30, 37]; when a *Plasmodium* agent reached specific points in its life-cycle, the vector or host agent would change infectious status.

One study characterized the infectious reservoir in humans according to parasite characteristics [68], with parasite densities known to be linked to host infectiousness. Studies investigated the impacts of infection not only on hosts, but also vector behaviours, such as altered biting rates [56, 57]. Parasite biology was also considered, including parasite strains [17, 74], PfHRP2 status [75, 76] and recrudescence of *P. vivax* malaria [30]; the latter model considered the disease to be a submodule of the overall simulation framework. Three models simulated multiple parasite clones [15, 17, 77], and four allowed antigenic parasite variation [15, 78–82], particularly to capture antimalarial resistance.

The choice of parasite, much like the *Anopheles* species, was often based on the dominant species in the target location. The dominance of *P. falciparum* malaria simulation reflects the attention paid to it, which is largely due to the historic relative burden of disease. Additionally, a number of interventions simulated were specific to *P. falciparum*, in particular the pre-erythrocytic RTS,S vaccine [14, 44, 83].

Modelling different *Plasmodium* species may require changes to model structure, for example to account for the recrudescence seen in *P. vivax* but not *P. falciparum* malaria. Pizzitutti et al. [30, 37] incorporated these differences by adding parameters governing recurrence time and risk of *P. falciparum*-triggered *P. vivax* recurrence. The parameters representing infection vary between *Plasmodium* species, based on prior evidence. Beyond these changes, the decision-tree model structures dictating the actions of agents were not changed.

### Environment and spatial modelling

Core environmental aspects related to vector activity were included, such as water sources for oviposition (egg-laying) [20], houses for blood meal locations [72], and meteorological data to account for seasonal patterns in transmission [58, 84]. Rainfall and temperature data were regularly used; model questions included the impact of wet season lengths [42] and hysteresis [40] on vector populations.

Thirty of the 90 models explicitly incorporated a spatial framework into their model (Fig. 1 and Additional file 2, column 11; explored further in Additional file 4). Seventeen models investigated malaria transmission in Africa, by simulating a specific location (e.g. [20, 85]) or using a hypothetical landscape representing a typical African village (e.g. [31, 53]). Two models represented transmission in the Amazon region [30, 37] and one each in Papua New Guinea [17], France [71], Haiti [86] and Bangladesh [63]. Spatial models also simulated local vector activity in a generic physical environment [51, 57, 58].

While many spatial models had detailed representations of a known landscape [25], this was not always the case, particularly with early spatial models [22, 86]. Three methods of spatial construction predominated: grid-based systems, patches and continuous landscapes. Additionally, Depinay et al. [58] explicitly linked vector locations to houses, but did not assign spatial coordinates, whilst Karl et al. [17] generated a probability of transmission based on the distance between host and vector locations to incorporate spatial biting factors.

In a grid-based landscape, the modelled area was most commonly divided into squares of a constant size, with elements of the landscape residing within a square (e.g. [22]). The HYDREMATS hydrology/entomology models were the exception, with grid size varying depending on proximity to human habitats, and complexity of water sources in the area [20]. There was interplay between the physical properties of the study area and the construction of the model. For example, the size of grid squares were related to parameters such as typical distance moved by mosquitoes in one time step, or larval habitat size [20]. The overall simulation area, typically a square between 600 m and 3000 m in length, was commonly justified based on *Anopheles* dispersal behaviour and typical village sizes being modelled [31]. Six models did simulate much larger areas [16, 17, 37, 62, 67, 85]; the last of these models used 1 km grids to describe the environment, yet characterized movement and interventions on much finer spatial scales [67]. Distances from which mosquitoes can target hosts or oviposition sites were guided by previous field data where possible [30, 31].

The additional spatial methods addressed opposing limitations of grid-based landscapes, namely total area and spatial precision. A patch framework was used by Silal et al. [16] and Eckhoff et al. [62] to represent areas of differing transmission characteristics (e.g. changing EIRs within districts), without explicitly modelling the environment. This method allowed for simulation of broader spatial areas. Rateb et al. [86] achieved this by representing Haiti as a set of “microenvironments” that host agents were impacted by. Conversely, models with a continuous landscape were used to allow variation in the location of houses, larval habitats, sugar sources and resting sites, and to model the impact of different physical environments [21, 31]. Each object had a specified location, and mosquitoes could sense objects within a certain circular distance, as opposed to a particular grid square. However, even when using a village-sized continuous landscape, models from Zhu et al. [21, 31, 36] still mapped random mosquito flight using a grid-based system, albeit on a 1 m × 1 m scale.

Some models also impute spatial results from non-spatial models by alternate means. The Malaria Atlas Project [87] and the Markham Seasonality Index [88] have generated methods to predict EIRs by location, and researchers have combined these tools with model outputs to generate maps of estimated disease burden across sub-Saharan Africa (SSA) [27, 54, 69, 78, 89, 90]. Models also used SSA rainfall data to replicate seasonality patterns [69, 91] and population estimates for host locations over large areas [91, 92]. In one instance, a spatial model of hypothetical locations was simulated in conjunction with geographic information systems (GIS) technology, to represent transmission in specific locations [61].

### Interventions

Fifty-eight studies assessed the impact of at least one intervention on malaria transmission, mosquito prevalence or EIR (Additional file 2, column 6). The majority of papers assessed multiple interventions, 21 assessed interventions in combination, and one investigated the removal of current malaria strategies [52]. Interventions could broadly be divided into those targeted at the human host (e.g. pharmacological) or the vector. Model structure tended to support simulation of the intervention being tested. For example, larval habitat elimination [51] or toxic sugar baits [21] were modelled spatially, requiring vectors as agents; whereas vaccination [18, 33, 44] and drug administration [93] studies involved human agents, in order to capture individual immune responses.

Interventions were included in models for two reasons: for assessment of effectiveness [18], or to replicate the ‘baseline’ characteristics of regional transmission [26], to allow additional interventions to be explored. In general, baseline scenarios included ITN [54] and “case management” [26], as defined by the current interventions in the location being modelled.

Simulation of interventions usually took one of three approaches. First, a specific intervention was defined to have a certain effect when in use, for example a medication having a fixed efficacy, and the impact on transmission is measured. Second, where environments were explicitly modelled, interventions were usually defined by their physical impact. For example, from a baseline scenario, an intervention might alter individual larval habitats [51] or remove sugar sources to reduce vector feeding [31], and simulations highlighted the change in the output of interest, such as disease burden or vector populations. Third, hypothetical interventions were described by their impacts, to target a specific aspect of malaria transmission [65]. The hypothetical impact may approximate a pre-existing intervention, such as halving human biting rates for a fixed period [94], or evaluate novel targets that may guide future control techniques.

### Other

The ease with which ABMs allow for heterogeneity and model complexity enables some less often considered transmission factors to be simulated. Examples include anthropophily (i.e. vector preference for human hosts) [19], sugar sources (e.g. fruit trees) [21, 31], travellers [16, 95], the impacts of larval densities in oviposition sites [59, 65], and the pharmacokinetics and pharmacodynamics of anti-malarials [75, 80, 91, 96].

## Data use, model outputs and analysis

### Parameter estimation and robustness of results

Most models used past literature or simulations to determine baseline parameter values (Additional file 2, column 8). Due to the nature of certain inputs, many parameters cannot or have not been estimated in field studies, and consequently authors used expert knowledge to select these values. Five papers provided no evidence that prior knowledge was used to estimate parameters. These models generally introduced a new modelling technique, or assessed hypothetical scenarios, as opposed to claiming accurate simulation of real-world situations.

Fifty-two papers used models that were either previously calibrated to data or presented calibration as a component of their work. Calibration techniques included the use of calibration vectors, least squares, maximum likelihood functions, and visual estimations. Twelve papers mentioned using Bayesian techniques for model fitting, with five providing credible intervals alongside point estimates of parameters [14, 23, 32, 50, 89, 91, 92, 97]. Of note, no papers calibrated all model parameters to data, which necessarily excludes certain parameter combinations that could produce accurate calibrations.

Validation was performed on models used in 31 papers. All major model frameworks were reported as validated. Validation techniques were rarely explained in detail. When described, validation was most commonly performed by running a calibrated simulation, and comparing model outputs to a dataset not used for calibration [98]. Methods of comparing models to data were rarely explained; use of a square distance function [37], log likelihoods [16] and docking techniques [99] were outlined. Successful model validation was often used to justify extending a model framework to include interventions or to assess their potential impact in the location of interest. A number of studies concluded that their model did not accurately fit the data used for validation [98], suggesting incomplete data may explain any discrepancies.

Forty-five papers either explicitly mentioned sensitivity analysis or described parameter variation and comparison of outputs. The techniques used were generally informal, with methods used rarely explained in detail, and reporting of results was uncommon. Where sensitivity analysis was explained, it involved altering calibrated baseline parameter values by a fixed percentage and assessing changes to outputs. Formal techniques employed included Latin Hypercube sampling [70, 72], regression tree analysis [72], and one-way and probabilistic sensitivity analysis [28].

### Optimization and cost-effectiveness analysis

A number of papers referenced optimization use, including in titles [21, 45, 85] and keywords [100], and seven papers performed analysis for the clear purpose of optimization (Additional file 2, column 16). Methods included changing the timing [94, 95], location [21] or implementation of interventions [45, 85]. Five studies assessed between nine and 144 scenarios each. In contrast, two studies described formal optimization methods [69, 97], whereby one or more objective functions are optimized using a defined search algorithm. By changing the combination and coverage of interventions in a variety of baseline settings, these studies compared 98,784 and 306,000 scenarios, respectively.

CEA was performed in 11 studies, with six of these using the OpenMalaria platform to assess various medical interventions [101–103] or pre-erythrocytic vaccine RTS,S/SA02A [26, 27, 104] (Additional file 2, column 14). CEA studies used known local costs of the physical intervention and the costs of implementation [27, 104] to determine the financial impact of interventions. These values were compared to benchmarks, such as the World Health Organization (WHO) standard of $150 per life-year gained [28], to determine the potential value of interventions. No studies investigated the maximum impact on outputs for a fixed cost, instead assessing cost-effectiveness of interventions for a specific level of impact.

When determining how to scale interventions for CEA, one method employed was incremental cost-effectiveness ratios (ICERs) [27, 103], in which a baseline scenario was compared to the introduction of a number of interventions, to determine the net cost per case averted of each method up to a certain threshold. Interventions are added iteratively in a manner that does not perfectly optimize their delivery but has the lowest additional cost for each new intervention block.

### Ensemble modelling and model comparison

Where multiple models analyse the same transmission scenario, ensemble modelling can be used to highlight robustness of results, or to assess the appropriateness of model structure. Similar to the role of sensitivity analysis in evaluating the sensitivity of model outputs to parameter values, ensemble modelling can assess aspects of both parametric and structural uncertainty. A precursor to this was performed by Ross et al., extending a baseline model to five variants to assess hypotheses regarding treatment and illness patterns [105]. In addition to the individual studies in this review, 12 publications included ensemble modelling of OpenMalaria model variants [106–117], and three studies performed “consensus modelling” across different frameworks [118–120] (see Additional file 5).

The OpenMalaria ensembles used from six to 14 model variants, selected from 30 tested alternatives. The 16 rejected models were either very similar to an accepted choice or could not be parameterized to the dataset used. For the three articles that compared outputs across the ensemble, the selected models were in agreement for most outputs [106, 107, 113]. The most common divergence was differing intervention efficacies predicted by models with in-built transmission heterogeneity, compared to those with uniform transmission levels between individuals. Interestingly, even though the fourteen models often reached the same conclusion, the estimated parameter values from calibration varied significantly across the ensemble [107].

The nine additional OpenMalaria ensembles aggregated the outputs from model variants, to investigate interventions such as vaccination [108, 116], seasonal malaria chemoprevention [115], mass test-and-treat strategies [109], and long-lasting insecticidal nets (LLINs) and IRS [110–112, 114]. One study predicted changes to disease outcomes and transmission upon the removal of vector control interventions from elimination programmes [117]. The aggregation of model outputs for each parameter set was performed to reduce the uncertainty arising from structural differences in models. In this way, the ensemble is considered as one model and its outputs interpreted as such.

Unlike the ensemble modelling above, when comparing inter-model variation, simulation outputs could not be aggregated. Despite the varied approaches, the consensus modelling largely drew consistent findings on the relationship between available malaria prevalence data and clinical incidence [118], and on the impacts of vaccination [119] and mass drug administration (MDA) [120].

## Discussion

Mathematical modelling plays an important role in malaria elimination, and agent-based approaches make a major contribution to these efforts. The extension of compartmental models to their early ABM equivalents arose from the need to understand malaria transmission at the individual level. The result is a rich array of model families and simulation techniques, adapted to a range of key issues in transmission and control.

In general, three core themes emerged regarding justification of ABM use. First, the greater importance of stochasticity in low-transmission settings, particularly settings approaching elimination, requires an alternative approach to traditional compartmental methods. Second, attempts to eliminate local transmission require discrete population simulations to incorporate spatially explicit environments at increasingly fine resolutions. Third, heterogeneities in disease progression and severity on the individual patient level result in varying efficacy of drug and vaccine interventions, which may be difficult to capture within a compartmental framework. These three arguments stem from a common point: a compartmental structure, based on averaging over a population, has limitations when that average does not adequately represent the individuals.

In addressing these issues, the benefits of agent-based techniques in this space are evident. Many papers in this review explicitly aimed to fill the knowledge gap regarding intervention use in low transmission environments. Most projects provided outputs robust at multiple transmission intensities, highlighting the flexibility of ABMs in low-prevalence settings. The HYDREMATS framework was used in multiple locations, incorporating environmental factors such as temperature and rainfall at different times, [25, 38, 39]. The OpenMalaria models progressed from assessing the force of infection of malaria transmission [32], to estimating cost-effectiveness of a vaccination programme [27]. Given the similarities of compartmental models of malaria to the original Ross–McDonald framework [6], it is clear that the depth and flexibility of agent-based methods are allowing new insights into malaria transmission and prevention.

The variation in the models described above highlights the difficulties in developing a standardized style of ABM for use in malaria epidemiology. However, this is arguably a major advantage, with the abundance of techniques allowing for the flexibility desired when transitioning from solely using compartmental models. Instead of suggesting a “gold standard” approach, it may be preferable to ensure the model style used is appropriate for the question at hand. For example, OpenMalaria’s early modelling of gametocyte densities did not use vector agents [18], but successfully provided insights into risks of fever, morbidity and mortality of patients [24]. The EMOD models initially described host-vector interactions without spatial consideration [19], but added this capability when required to assess interventions [62, 85]. Therefore, while not every model incorporated every aspect of malaria epidemiology, each was tailored to the research question at hand.

Conversely, if modelling groups are considering extending their model frameworks, particularly to influence policy, there is potential to draw from the features of one another. For example, HYDREMATS currently includes human and mosquito agents, while the characteristics of human infection are more detailed in the OpenMalaria simulations. Therefore, the time variability of individual gametocyte density, probabilities of fever, morbidity and mortality, and the infectivity of hosts to vectors used in the OpenMalaria framework could be adapted into HYDREMATS to more realistically replicate disease transmission. However, in neither of these simulations do humans move, whereas this process is explicitly simulated in Zhu et al. [31] and Pizzitutti et al. [37] to better represent vector-host feeding patterns. Pizzitutti et al. [30] and EMOD [19] include human behavioural reactions to biting rates (i.e. time-dependent intervention use) and the probabilities of successful blood meals, respectively. These five models have components that simulate vector, egg and human populations, effects of climate on larval habitats, anthropophily, ITN, IRS, larval habitat removal, vaccination, anti-malarial use, attractive toxic sugar baits, and rates of human disease. As each framework provides insights into key components of malaria transmission, all of which are important in guiding elimination strategies.

To some extent, combining model structures across research teams can be considered an extension of the use of “submodules” already undertaken by larger modelling frameworks. The HYDREMATS team have successfully integrated detailed larval habitat and entomologic models [55], and OpenMalaria now includes upwards of seven modules of human disease states and interventions [100]. Modular projects such as HYDREMATS, EMOD [85] and OpenMalaria have provided insights into transmission dynamics, vector populations, disease severity, and the contributors to these factors. Given the importance of comprehensive modelling to guide policy decisions, the potential for combining the strengths of validated models to enhance decision-making capabilities of ABMs could be explored.

A key target for modelling low-transmission settings is a focus on spatial representation and heterogeneity. ABMs can shift spatial heterogeneity from a typically “patch-based” compartmental framework into a continuous space, by having explicit locations for environmental objects, dwellings and agents. These detailed descriptions of the landscape are coupled with local knowledge of physical characteristics (such as host/vector movement patterns) to simulate malaria transmission, ecology, and the impact of interventions based on their location. These insights include the distances between larval habitats and houses to effectively reduce malaria transmission [53], and the impact on systematic versus random location of attractive toxic sugar baits (ATSB) on mosquito abundance [21]. This style of intervention inherently requires spatial modelling, although interventions such as ITNs and IRS have been modelled in both spatial and non-spatial simulations. Examining these interventions in physical environments allows the impact of factors such as vector movement and the proximity of unprotected individuals to be measured.

Potential extensions to spatial models include varying elimination strategies across a landscape, and increasing the size of the geographical area modelled. For example, consider a small community near a local water source, and a nearby larger population with better access to healthcare. A model could implement larval source management at the local water sources, whilst increasing access to vaccines and anti-malarials in the healthcare centre. Human movement dynamics [121] could be incorporated to assess the relative effectiveness of each interventions across both populations. This style of modelling may more accurately represent the manner in which interventions are implemented at the local level. Regarding model areas, most simulations of real-world environments only covered the area of a specific village, with sizes ranging from 600 m × 600 m to 3000 m × 3000 m. These spatial ranges have been limited by computational power, but this limitation will continue to decrease over time. There also may be a lack of access to consistent geospatial data over larger areas; however, many models only included spatial data on physical habitats, which would be collected in a similar manner over larger areas. If it is deemed useful to model malaria over a wider area, techniques from other fields may be used, such as probability modelling of invasive species, which has been performed for an area of over 35,000 km^2^ [122].

Regarding the locations of malaria modelling, there is an understandable focus on SSA, which was responsible for 88% of the global malaria burden in 2015 [2]. However, there has also been a recent increase in attention on South-East Asia (SEA), which is responsible for 10% of global cases and has emerging issues with drug resistance [2]. Moreover, challenges such as artemisinin resistance and insecticide resistance are more prominent in these areas. Despite this, approximately half of all malaria cases outside Africa in 2015 were due to *P. vivax* [2], while *Plasmodium knowlesi* malaria transmission is increasing in locations, such as Malaysia [123]. The methods of ABM construction used in SSA and SEA, and for *P. falciparum* malaria, suggest transferability to other regions and *Plasmodium* species, which will be important as data availability from these areas improves and attention turns to global elimination.

Many ABMs reviewed here had an emphasis on informing policy and explicitly aimed to understand specific programmatic questions (e.g. [16, 28, 78, 83]). To reliably inform public health decisions, there must be confidence in the assumptions guiding model creation, in particular regarding choice of parameter values. The methods for estimating key parameters varied greatly across the literature. Parameter justification was not always clear [13, 22, 51, 65]; when explained, models generally calibrated a range of parameters to existing data, or provided references for their choice of fixed values. Further, no previous models have calibrated all parameters to data. Importantly, ensemble modelling of the OpenMalaria variants calibrated 14 variants to the same dataset, but parameter values varied significantly across between models [107]. Given that uncertainty remains even after calibration, it is important to apply a systematic and comprehensive approach to parameter estimation before using models for predicting parameter impact or forecasting.

Whilst field data exists for a range of parameters, researchers must be pragmatic about the possibility of adequately calibrating complex ABMs, particularly when data is required in resource-poor settings. The fixing of well-established values can reduce the parameter space to be searched using calibration methods. Alternatively, techniques such as Markov chain Monte Carlo (MCMC) can search the entire parameter space, or more precisely that part of parameter space that has non-negligible posterior probability. MCMC has already been used in malaria ensemble modelling [108, 116, 118] and parameter estimation by Griffin et al. [14], as well as in modelling of other infectious diseases [124, 125]. Approaches such as MCMC and approximate Bayesian computation are increasing in popularity as including uncertainty in model parameters becomes more common [126].

An arguably more pressing area of need for development is optimization, with methods for agent-based models still in their infancy. In this review, studies that reported optimization usually simulated a suite of different interventions, or the same interventions at different levels of coverage or timing. Cost-effectiveness analysis was typically approached in the same manner. Whilst conclusions were provided as to the most effective simulation approach, true optimization was rare [69, 97], using formal techniques to identify parameter values that optimize one or more objective functions. Given the role of ABMs in modelling interventions in low-transmission settings, formal optimization techniques are important for enhancing the ability of models to guide policy.

Deterministic models have already been used as the basis of optimization of interventions for various infection diseases [125, 127, 128]. Strategies for the optimization of interventions within ABMs appear less common, possibly due to the high computational burden of finding consistent minima in the presence of stochasticity. It is difficult to define how to best approach optimization from an agent-based standpoint. A systematic review of ABMs for optimization problems [129] highlighted techniques used for disciplines such as scheduling, supply chain management, energy systems planning and transportation and logistics. As is likely the case regarding spatial methods, optimization of malaria transmission modelling (and infectious disease simulation more broadly) may benefit from adapting approaches outside the field to a new context.

Increased clarity in model reporting would be of great benefit to both the creators of ABMs and their audience. While many papers included detailed supplementary materials for additional results, project descriptions and calibration, validation, sensitivity analysis and optimization techniques, the intricacies of these techniques often unclear. A protocol exists for the description of ABMs [130], and models that used it [21, 31, 76] were simple to understand and appeared easily replicable by external groups. Further transparency includes sharing of the mathematics and code [76] of models. These small steps in documentation would allow for increased verification and validation of models, as well as increasing opportunities for collaboration between modelling groups.

Beyond individual models, ensemble modelling is an important tool for generating robust conclusions about malaria transmission. A review of ebola models advocated for an ensemble modelling approach that adequately compares state-of-the-art models, but also allows for model diversity [131]. For malaria models built for similar purposes (for example, to estimate certain parameters of interest, or to predict the value of an intervention), both inter- and intra-model comparison has been conducted. In some cases, two or three interventions have been simultaneously assessed using this approach [110, 114, 115]. The next steps for ensemble and consensus modelling may include more interventions, spatial modelling, and developing techniques to determine which models are most appropriate when ensemble members differ in parameter estimates or outputs.

## Conclusions

As malaria transmission continues to decline and interventions become more nuanced, agent-based modelling will have an increasingly important role to play in elimination programmes. A variety of techniques have already been developed, and models are increasingly tailored to the question of interest. The flexibility of ABMs is a key feature, with progressive model extensions and fine-grain spatiotemporal simulation two clear examples.

The breadth of model frameworks makes it difficult to develop guidelines for ABM construction, but this should be considered a strength. The correct approach to agent-based modelling is likely a “horses for courses” approach, whereby the question at hand guides the development of the model; current approaches may be used to guide such choices. As models transition from tools for analysing an epidemic to guiding policy directions, it is important to be aware of the current literature and techniques available. Existing model frameworks cover many transmission factors, so cross-collaboration may bring about larger models that can simulate many interventions and provide outputs regarding vector populations, host disease progression and the success of elimination strategies. However, using larger models may create difficulties with simulation. Improved techniques in parameter estimation and optimization could enhance the role ensemble modelling currently plays in evaluating interventions for specific geographical and transmission contexts.

With the ever-increasing computing power available to researchers, detailed ABMs that accurately reflect the biology of malaria transmission are increasingly feasible on a fine spatial resolution over large geographical regions. As such, agent-based modelling will be an important tool for helping to inform malaria elimination strategies over the coming years.

## Additional files

**Additional file 1.** Systematic review search strategies.

**Additional file 2.** Key characteristics of individual-based models of malaria.

**Additional file 3.** Adapted PRISMA search flow diagram of study selection.

**Additional file 4.** Overview of elements of spatial models.

**Additional file 5.** Overview of ensemble and comparative models.

## Abbreviations

ABM: agent-based model
SSA: sub-Saharan Africa
ITN: insecticide-treated bed nets
ACT: artemisinin-based combination therapy
IRS: indoor residual spraying
GMEP: Global Malaria Eradication Programme
NMCP: National Malaria Control Programme
CEA: cost-effectiveness analysis
EIR: entomological inoculation rate
WHO: World Health Organization
MDA: mass drug administration
ICER: incremental cost-effectiveness ratio
LLIN: long-lasting insecticidal net
SEA: South-East Asia
MCMC: Markov chain Monte Carlo
