# Supplementary material for: Agent-based models of malaria transmission: a systematic review
Source: Malar J. 2018 Aug 17;17:299. doi: 10.1186/s12936-018-2442-y (PMC6098619; doi:10.1186/s12936-018-2442-y)
Supplement: Supplementary file 1 — Additional file 1. Systematic review search strategies. [file 12936_2018_2442_MOESM1_ESM.docx]

# Additional Information - Agent-Based Models of Malaria Transmission: A Systematic Review

Smith, Neal R.; Trauer, James M.; Gambhir, Manoj; Richards, Jack S.; Maude, Richard J.; Keith, Jonathan M.; Flegg, Jennifer A.

## Additional File 1: systematic review search strategies

### OVID MEDLINE review search strategy

Review performed on March 03, 2017 (updated May 25, 2018). See <http://resourcecenter.ovid.com/site/help/documentation/ospa/en/Content/advanced.htm> for definitions of search operations.

1. exp Malaria/

2. exp Malaria Vaccines/

3. exp Plasmodium/

4. exp Antimalarials/

5. exp Artemisinins/

6. exp Fluorenes/

7. exp Ethanolamines/

8. exp Cinchona Alkaloids/

9. exp Aminoquinolines/

10. exp Pyrimethamine/

11. exp Proguanil/

12. exp Sulfonamides/

13. exp Mefloquine/

14. exp Atovaquone/

15. exp Doxycycline/

16. exp Lincomycin/

17. exp Culicidae/

18. malari*.mp.

19. Plasmodi*.mp.

20. antimalari*.mp.

21. Artemisinin*.mp.

22. Fluoren*.mp.

23. Ethanolamin*.mp.

24. Cinchon*.mp.

25. Quinidin*.mp.

26. Quinin*.mp.

27. Aminoquinolo*.mp.

28. Chloroquinolo*.mp.

29. Amodiaquin*.mp.

30. Primaquin*.mp.

31. Pyrimethami*.mp.

32. Proguan*.mp.

33. Sulfonamid*.mp.

34. Mefloquin*.mp.

35. Atovaquo*.mp.

36. Halofantri*.mp.

37. Doxycyc*.mp.

38. Clindamyc*.mp.

39. Lincomyc*.mp.

40. Mosquit*.mp.

41. Culicid*.mp.

42. Anophel*.mp.

43. Chloroquin*.mp.

44. 1 or 2 or 3 or 4 or 5 or 6 or 7 or 8 or 9 or 10 or 11 or 12 or 13 or 14 or 15 or 16 or 17 or 18 or 19 or 20 or 21 or 22 or 23 or 24 or 25 or 26 or 27 or 28 or 29 or 30 or 31 or 32 or 33 or 34 or 35 or 36 or 37 or 38 or 39 or 40 or 41 or 42 or 43

45. exp Epidemiology/

46. exp Demography/

47. exp Disease Outbreaks/

48. exp Disease Transmission, Infectious/

49. exp Epidemiologic Measurements/

50. exp Epidemiologic Methods/

51. Epidemio*.mp.

52. Disease Outbreak*.mp.

53. Disease Transmi*.mp.

54. Epidemic*.mp.

55. Endemic*.mp.

56. Pandemic*.mp.

57. reproductive number*.mp.

58. incubation period*.mp.

59. infectious period*.mp.

60. latent period*.mp.

61. case fatality*.mp.

62. demograph*.mp.

63. contact trac*.mp.

64. monito*.mp.

65. survei*.mp.

66. 45 or 46 or 47 or 48 or 49 or 50 or 51 or 52 or 53 or 54 or 55 or 56 or 57 or 58 or 59 or 60 or 61 or 62 or 63 or 64 or 65

67. 44 and 66

68. Agent base*.mp.

69. Individual base*.mp.

70. Microsim*.mp.

71. 68 or 69 or 70

72. 44 and 71

73. 66 and 71

74. 67 and 71

### OVID Embase review search strategy

Review performed on March 12, 2017 (updated May 23, 2018). See <http://resourcecenter.ovid.com/site/help/documentation/ospa/en/Content/advanced.htm> for definitions of search operations.

1. exp malaria/

2. exp malaria vaccine/

3. exp malaria control/

4. exp malaria rapid test/

5. exp Plasmodium/

6. exp antimalarial agent/

7. exp antimalarial drug susceptibility/

8. exp antimalarial activity/

9. exp fluorene derivative/

10. exp ethanolamine derivative/

11. exp ethanolamine derivative/

12. exp sulfonamide/

13. exp doxycycline/

14. exp doxycycline hyclate/

15. exp aminoglycoside antibiotic agent/

16. exp mosquito/

17. exp mosquito bite/

18. exp bed net/

19. exp insect repellent/

20. Malari*.mp.

21. plasmodi*.mp.

22. antimalari*.mp.

23. Mosquit*.mp.

24. Culicid*.mp.

25. Anophel*.mp.

26. antiplasmod*.mp.

27. naphthoquin*.mp.

28. quinaz*.mp.

29. Quinol*.mp.

30. fluoroadenosin*.mp.

31. acridon*.mp.

32. trichlorophenoxy.mp.

33. norprimaq*.mp.

34. acridin*.mp.

35. methoxyquin*.mp.

36. hydroxychlor*.mp.

37. albitiazolium.mp.

38. Amodiaq*.mp.

39. apicidi*.mp.

40. amopyroq*.mp.

41. Aminoquinolo*.mp.

42. arteeth*.mp.

43. artefenom*.mp.

44. artefl*.mp.

45. artelin*.mp.

46. artemet*.mp.

47. artemis*.mp.

48. arterol*.mp.

49. artesun*.mp.

50. atovaquo*.mp.

51. azithromy*.mp.

52. benflumet*.mp.

53. bulaqu*.mp.

54. chloroquin*.mp.

55. Chlorprog*.mp.

56. Cinchon*.mp.

57. cipargam*.mp.

58. clindamyc*.mp.

59. collagena*.mp.

60. dabequ*.mp.

61. debutylhal*.mp.

62. deethylamod*.mp.

63. deethylchlo*.mp.

64. deoxyartem*.mp.

65. Dideethylchlo*.mp.

66. dihydroarte*.mp.

67. Doxycyc*.mp.

68. cycloguan*.mp.

69. Enpiroli*.mp.

70. Fansime*.mp.

71. Ferroqui*.mp.

72. Floxacri*.mp.

73. Fluoren*.mp.

74. Gedunin*.mp.

75. Girolli*.mp.

76. Halofantri*.mp.

77. Hydroxypip*.mp.

78. isopentaqu*.mp.

79. Lapacho*.mp.

80. Lincomyc*.mp.

81. Mefloquin*.mp.

82. Menocton*.mp.

83. Mepacri*.mp.

84. Metakel*.mp.

85. benzylaminopropyl.mp.

86. dimethoxyphenethyl.mp.

87. pamaqui*.mp.

88. pentaqui*.mp.

89. Phaeanth*.mp.

90. Piperaq*.mp.

91. Primaquin*.mp.

92. Proguan*.mp.

93. pyrimethami*.mp.

94. Pyronarid*.mp.

95. Quinim*.mp.

96. Quinin*.mp.

97. Tafenoq*.mp.

98. Tebuq*.mp.

99. Tilbroq*.mp.

100. Tiliq*.mp.

101. Sontoq*.mp.

102. sulfonamid*.mp.

103. Acedapso*.mp.

104. Dapso*.mp.

105. quinacr*.mp.

106. Quinid*.mp.

107. Sulfadox*.mp.

108. Sulfale*.mp.

109. trimetho*.mp.

110. qinghao*.mp.

111. 1 or 2 or 3 or 4 or 5 or 6 or 7 or 8 or 9 or 10 or 11 or 12 or 13 or 14 or 15 or 16 or 17 or 18 or 19 or 20 or 21 or 22 or 23 or 24 or 25 or 26 or 27 or 28 or 29 or 30 or 31 or 32 or 33 or 34 or 35 or 36 or 37 or 38 or 39 or 40 or 41 or 42 or 43 or 44 or 45 or 46 or 47 or 48 or 49 or 50 or 51 or 52 or 53 or 54 or 55 or 56 or 57 or 58 or 59 or 60 or 61 or 62 or 63 or 64 or 65 or 66 or 67 or 68 or 69 or 70 or 71 or 72 or 73 or 74 or 75 or 76 or 77 or 78 or 79 or 80 or 81 or 82 or 83 or 84 or 85 or 86 or 87 or 88 or 89 or 90 or 91 or 92 or 93 or 94 or 95 or 96 or 97 or 98 or 99 or 100 or 101 or 102 or 103 or 104 or 105 or 106 or 107 or 108 or 109 or 110

112. exp epidemiology/

113. exp epidemiological data/

114. exp epidemiological monitoring/

115. exp demography/

116. exp epidemic/

117. exp endemic disease/

118. exp pandemic/

119. exp disease transmission/

120. Epidemiolo*.mp.

121. Disease outbreak*.mp.

122. Disease Transmi*.mp.

123. Endemic*.mp.

124. Epidemic*.mp.

125. Pandemic*.mp.

126. Reproductive number*.mp.

127. Incubation period*.mp.

128. Infectious period*.mp.

129. Latent period*.mp.

130. case fatality*.mp.

131. demograph*.mp.

132. contact trac*.mp.

133. monitor*.mp.

134. surveil*.mp.

135. inciden*.mp.

136. prevalen*.mp.

137. mortal*.mp.

138. infection rate.mp.

139. 112 or 113 or 114 or 115 or 116 or 117 or 118 or 119 or 120 or 121 or 122 or 123 or 124 or 125 or 126 or 127 or 128 or 129 or 130 or 131 or 132 or 133 or 134 or 135 or 136 or 137 or 138

140. Agent base*.mp.

141. Individual base*.mp.

142. microsim*.mp.

143. 140 or 141 or 142

144. 111 and 139

145. 111 and 143

146. 139 and 143

147. 111 and 139 and 143

### EBSCO CINAHL Plus review search strategy

Review performed on March 12, 2017 (updated May 15, 2018). See <https://help.ebsco.com/interfaces/CINAHL_MEDLINE_Databases/Training_Promotion/CINAHL_Databases_Advanced_Searching_Tutorial> for definitions of search operations.

1. (MH "Malaria")

2. (MH "Antimalarials+")

3. (MH "Ethanolamines+")

4. (MH "Alkaloids+")

5. (MH "Pyrimethamine")

6. (MH "Chloroguanide")

7. (MH "Mefloquine")

8. (MH "Atovaquone")

9. (MH "Doxycycline")

10. (MH "Lincomycin+")

11. (MH "Mosquitoes")

12. (MH “Mosquito Nets”)

13. (MH “Mosquito Vectors”)

14. malari*

15. plasmodi*

16. antimalari*

17. Artemisinin*

18. Flouren*

19. Ethanolamin*

20. cinchon*

21. Quinidin*

22. Quinin*

23. Aminoquinolo*

24. Primaquin*

25. Proguan*

26. Sulfonamid*

27. Mefloquin*

28. Atovaquo*

29. Halofantri*

30. Doxycyc*

31. Clindamyc*

32. Lincomyc*

32. Mosquit*

34. Culicid*

35. Anophel*

36. Chloroquin*

37. S1 OR S2 OR S3 OR S4 OR S5 OR S6 OR S7 OR S8 OR S9 OR S10 OR S11 OR S12 OR S13 OR S14 OR S15 OR S16 OR S17 OR S18 OR S19 OR S20 OR S21 OR S22 OR S23 OR S24 OR S25 OR S26 OR S27 OR S28 OR S29 OR S30 OR S31 OR S32 OR S33 OR S34 OR S35 OR S36

38. (MH "Epidemiology+")

39. (MH "Demography+")

40. (MH "Disease Outbreaks")

41. (MH "Disease Transmission")

42. (MH "Disease Transmission, Horizontal+")

43. (MH "Disease Transmission, Vertical")

44. (MH "Epidemiological Research+")

45. Epidemio*

46. disease outbreak*

47. disease transmi*

48. epidemic*

49. endemic*

50. pandemic*

51. reproductive number*

52. incubation period*

53. infectious period*

54. latent period*

55. case fatality*

56. demograph*

57. contact trac*

58. monito*

59. survei*

60. S38 OR S39 OR S40 OR S41 OR S42 OR S43 OR S44 OR S45 OR S46 OR S47 OR S48 OR S49 OR S50 OR S51 OR S52 OR S53 OR S54 OR S55 OR S56 OR S57 OR S58 OR S59

61. agent base*

62. agent-base*

63. individual base*

64. individual-base*

65. microsim*

66. S61 OR S62 OR S63 OR S64 OR S65

67. S37 AND S60

68. S37 AND S66

69. S60 AND S66

70. S37 AND S60 AND S66
