# Supplementary material for: Agent-based models of malaria transmission: a systematic review
Source: Malar J. 2018 Aug 17;17:299. doi: 10.1186/s12936-018-2442-y (PMC6098619; doi:10.1186/s12936-018-2442-y)
Supplement: Supplementary file 2 — Additional file 2. Key characteristics of individual-based models of malaria. [file 12936_2018_2442_MOESM2_ESM.docx]

# Additional Information - Agent-Based Models of Malaria Transmission: A Systematic Review

Smith, Neal R.; Trauer, James M.; Gambhir, Manoj; Richards, Jack S.; Maude, Richard J.; Keith, Jonathan M.; Flegg, Jennifer A.

## Additional File 2: Key characteristics of individual-based models of malaria.

### Columns defined as follows. *Agents*: denotes Individuals within a population that have individual characteristics and are traceable throughout a simulation; H – human agents, M – mosquito agents. *Plasmodium*: Y – specific *Plasmodium* species simulated. *Intervention*: Y – interventions modelled and compared to control simulation. *Environment*: Y – environmental considerations included in model. *Parameters*: P – data from previous studies used to estimate at least one parameter, C – at least one parameter from model estimated using calibration techniques, V – model validated using alternative dataset, S – sensitivity analysis performed, by altering the value of at least one parameter to determine impact on an outcome of interest. *Key inputs* and *Key outputs*: the most relevant inputs and outputs of the model for the simulation being described. *Spatial*: Y – model includes spatial representation of physical environment. *Age-stratification*: Y – humans are stratified by age for results or model parameters. *Cost analysis*: Y – interventions are costed within model. *Other notable model features*: as described. *Intervention Optimisation*: Y – Paper includes statement of intent to maximise the effectiveness of interventions within the simulation. Other notation within table: †Where ABMs were developed from other model types, the original model is listed above it, without further details in table. *Paper provides overview of model structure in which these elements are not clear. **Spatial construction outlined but not mathematically present. ‡Model outlines capacity for intervention modelling, but simulation not performed.

| **Model** | | **Purpose** | **Agents** | **Plasmodium** | **Interventions** | **Environment** | **Parameters** | **Key Inputs** | **Key Outputs** | **Spatial** | **Bayesian** | **Age stratification** | **Cost Analysis** | **Other notable model features** | **Optimisation** | **Reference** |
| --- | --- | --- | --- | --- | --- | --- | --- | --- | --- | --- | --- | --- | --- | --- | --- | --- |
| **Original** | **Adapted** |  |  |  |  |  |  |  |  |  |  |  |  |  |  |  |
| McKenzie (1998) | | Introduce discrete simulation model, compare results to compartmental approaches | H,M | Y | N | N | P | Intrinsic and extrinsic incubation, host infectious period, host immunity | Vector population size, host malaria prevalence | N | N | N | N | Array-based simulation | N | (1) |
|  | McKenzie (2001) | Assess impact of varying vector populations on parasite extinction | H,M | Y | N | N | P | Intrinsic and extrinsic incubation, host infectious period, host immunity | Malaria prevalence, vector population size, rate of parasitic extinction | N | N | N | N | Seasonal variation in vector population size | N | (2) |
|  | McKenzie (2005) | Embed within-host parasite-immune system dynamic model within ABM | H,M | Y | N | N | P,C,S | Gametocyte decay rate, recombination rate, human population turnover, infection parameters | Frequency of establishment, time to parasite extinction | N | N | N | N | Replacement of hosts within naïve individuals | N | (3) |
| Gu (2003) | | Develop object-oriented design for ABM of *P. falciparum* malaria transmission | H,M | Y | Y | N | P,C | Human biting rate (HBR) | Scenario-specific probability of malaria elimination | N | N | Y | N | Natural immunity, population migration | Y | (4) |
| Gu (2009A) | | Create guidelines for programs of environmental management by larval source reduction | H,M | N | Y | Y | P | None | Total mosquito abundance, HBR, entomological inoculation rate (EIR), malaria incidence and prevalence | Y | N | N | N | Various village housing layouts | N | (5) |
|  | Gu (2009B) | Assess effects of insecticide treated nets (ITNs) on malaria transmission | H,M | N | Y | Y | P,S | Outcome parameters | Mosquito populations, EIR, malaria incidence | Y | N | N | N | Various village housing layouts | N | (6) |
| Depinay (2004) | | Create spatial ABM of *Anopheles* life cycle incorporating biological and environmental factors, present usefulness of model in hypothetical simulation | H,M | N | Y | Y | P,C,S | Temperature, moisture, number of water pools | Number of mosquitoes | Y^**^ | N | N | N | Adult aestivation, predators and pathogens of *Anopheles* mosquitoes | N | (7) |
| Molineaux (2001) | |  |  |  |  |  |  |  |  |  |  |  |  |  |  | (8) |
|  | Dietz (2006) | Calibrate model of first wave of parasitaemia, estimate vaccine efficacy | H | Y | Y | N | C | Asexual parasite densities, immune parameters | Vaccine efficacy | N | N | N | N | Immune responses | N | (9) |
| Rateb (2005) | | Model impact of hypothetical malaria education on malaria transmission in Haiti | H | N | Y | Y | P | Disease parameters, number of education and health centres | Average education level, ratio of infected individuals, mortality ratio | Y | N | Y | N | Human breeding, education and hospital location | N | (10) |
| Smith (2006A) | | Overview of multi-purpose dynamical model, particularly regarding vaccine efficacy | H | Y | N | N | * | * | * | N | N | Y | N |  | * | (11) |
|  | Smith (2006B) | Model relationship between EIR for *P. falciparum* malaria and force of infection in endemic areas | H | N | Y | N | P,C,V | EIR | Survival probabilities, force of infection | N | Y | Y | N | Natural immunity Liver stages of parasite | N | (12) |
|  | Maire (2006A) | Assess effects of acquired immunity on parasite densities | H | Y | N | N | P,C,V | EIR | Parasite densities by age | N | N | Y | N | Natural immunity | N | (13) |
|  | Ross (2006A) | Describe relationship between host infectivity and *P. falciparum* asexual parasite density in the human host | H | Y | N | N | P,C,V,S | EIR | Host infectivity | N | Y | Y | N | Sex of gametocytes | N | (14) |
|  | Killeen (2006) | Validate model from Ross (2006A) against data from Africa and Papua New Guinea | H | Y | N | N | P,C,V | EIR | Mean infectiousness of human population to mosquitoes | N | N | Y | N |  | N | (15) |
|  | Smith (2006C) | Predict pyrogenic thresholds of acute illness in *P. falciparum* malaria | H | N | N | N | P,C | EIR | Incidence of clinical malaria; individual pyrogenic thresholds | N | N | Y | N | Pyrogenic thresholds | N | (16) |
|  | Ross (2006B) | Assess malaria-attributable morbidity and mortality, model age-specific patterns in morbidity and mortality | H | Y | N | N | P,C | Relative incidence of severe malaria, entomologic data, malaria-specific mortality rates | Incidence of severe malaria, episodes of clinical malaria, death rates | N | N | Y | N | Natural immunity co-morbidities | N | (17) |
|  | Tediosi (2006A) | Predict incidence of clinical episodes and mortality, assess effects of case management | H | Y | Y | N | P,C,S | EIR | Death, sequelae, parasites, disability-adjusted life years/ years of life lost (DALYs/YLLs), treatment costs per capita | N | N | Y | Y | Health care system capacities, decision tree approach to treatment | N | (18) |
|  | Maire (2006B) | Validate Smith (2006A) against vaccine trial data | H | Y | Y | N | C,V | EIR | Time to first episode of clinical malaria | N | N | Y | N |  | N | (19) |
|  | Maire (2006C) | Predict effects of RTS,S/AS02A vaccine in expanded program on immunisation (EPI) in sub-Saharan Africa (SSA) | H | Y | Y | N | P,C,V,S | EIR, vaccine profile parameters | Uncomplicated cases, severe cases, mortality, patent parasitaemia, anaemia, cumulative vaccine effectiveness | N | N | Y | N | Acquired immunity | N | (20) |
|  | Tediosi (2006B) | Assess cost-effectiveness of adding RTS,S/AS02 vaccine into EPI | H | Y | Y | N | P,S | Predicted cost per dose delivered, cost per fully immunized child (FIC) | DALYs, YLL, direct costs, productivity gains | N | N | N | Y |  | N | (21) |
|  | Smith (2008) | Overview of extensions to Smith (2006A) model | H | Y | Y^‡^ | Y | * | * | * | N | N | * | * | * | * | (22) |
|  | Ross (2008) | Investigate potential mechanisms of action of IPT in infants (IPTi), predict likely impact on morbidity and mortality | H | Y | Y | N | P,C,V | Transmission intensity, treatment coverage | Clinical episodes | N | N | Y | N | *dhfr* mutations that impact treatment failure | N | (23) |
|  | Penny (2008) | Predict population effects of malaria vaccines on transmission, morbidity, mortality | H | Y | Y | N | P,C | EIR, vaccine delivery modality and coverage, vaccine properties | Proportion of vectors infected at each feed, time to local elimination, severe cases and deaths averted | N | N | Y | N | Multiple vaccine delivery modalities and vaccine types | N | (24) |
|  | Tediosi (2009) | Cost-effectiveness analysis (CEA) of varying vaccine types, prices and delivery modalities | H | Y | Y | Y | P,S | Vaccine delivery costs, case management costs | Cost per uncomplicated episode, severe episode, and DALY averted | N | N | N | Y | Delivery modalities, varied vaccine costs | N | (25) |
|  | Ross (2010) | Simulate effects of heterogeneity on malaria burden, age-prevalence and incidence | H | Y | Y | N | P,C,S | EIR, treatment-seeking rate | Age-curves for parasite prevalence, uncomplicated and severe episodes, direct and indirect mortality,treatment episodes, hospital admissions | N | N | Y | N | Natural immunity, episode severity, infectivity to mosquitoes | N | (26) |
|  | Ross (2011) | Determine cost-effectiveness of intermittent preventive treatment for malaria in infants and children | H | Y | Y | N | P,C,V,S | Cost of interventions, target age group, delivery channel, seasonal or year- round delivery, transmission intensity, seasonality, timing of first IPT dose for seasonal delivery, coverage levels of treatment | DALYs averted, cost per DALY averted, transmission intensity | N | N | Y | Y | Drug resistance | N | (27) |
|  | Maire (2011) | Analysis of uncertainties in vaccine cost-effectiveness | H | Y | Y | N | P,C,S | Age-prevalence | Incremental cost-effectiveness ratios (ICERs), expected value of perfect information | N | Y | Y | Y | Transmission levels, expected value of perfect information | N | (28) |
|  | Chitnis (2012) | Incorporate seasonality and heterogeneous humans into pre-existing model | H,M | Y | Y | Y | P,C | Probabilities and rates of particular outcomes in the transmission process | EIR, malaria clinical incidence and prevalence | N | N | N | N | Removal of interventions | N | (29) |
|  | Crowell (2013) | Predict incremental cost-effectiveness of MSAT campaigns | H | Y | Y | N | P,S | Pre-intervention EIR, MSAT timing, coverage and compliance, costing of MSAT | Parasite prevalence, episodes averted, incremental cost-effectiveness ratio | N | N | Y | Y | Incremental cost-effective ratios for CEA | N | (30) |
| Richard (1993) | |  |  |  |  |  |  |  |  |  |  |  |  |  |  | (31) |
|  | Gurarie (2007) | Present stochastic model of infection, examine effects of drug treatment and vaccination of children | H | N | Y | N | Nil | EIR | Immune effector variable, severity index, severity count, individual infectious state prevalence | N | N | Y | N | Maternal immunity | N | (32) |
| Gurarie (2012) | | Develop in-host model of parasite densities, validate through comparing ABM-predicted malaria prevalence to field observations | H | Y | N | N | P,C,V,S | EIR | Malaria prevalence, number of infected RBCs per µL of blood (individual) | N | N | N | N | Merozoite invasion of RBCs, antigenic variation of parasites | N | (33) |
| Linard (2008) | | Simulate variations in contact rate between potential malaria vectors and hosts in the Camargue | H,M | N | N | Y | P,S | Mosquito development parameters, protection rates, average daily temperature | Actual biting rate | Y | N | N | N | Horse and cattle agents, seasonally-dependent tourist numbers | N | (34) |
| Bomblies (2008) | | Calibration and validation of hydrology and entomology models embedded into an ABM of malaria transmission | H,M | N | N | Y | P,C,V,S | Hydrology and entomology data from the Sahel | Vectorial capacity, EIR, malaria prevalence, live mosquito numbers | Y | N | N | N | CO_2_-seeking behaviour, land surface scheme, predation of younger larvae by older ones | N | (35) |
|  | Bomblies (2009A) | Simulate village-scale *Anopheles* mosquito population dynamics in two locations | H,M | Y | N | Y | P,C,V,S | Pool water temperatures, water source locations, topography, vegetation, soil types | Water coverage, water depth, mosquito abundance | Y | N | N | N | Overland flow routing component | N | (36) |
|  | Bomblies (2009B) | Assess impact of hypothetical climate change patterns on malaria transmission | H,M | N | N | Y | P,C,V,S | Precipitation, temperature, humidity, radiation, wind speed and direction | Pool depth, mosquito abundance, vectorial capacity | Y | N | N | N |  | N | (37) |
|  | Stryker (2012) | Investigate the effects of land use change on hydrological processes impacting mosquito abundance | H,M | N | N | Y | P,C,V,S | Precipitation, temperature, humidity, radiation, wind speed and direction | Percentage volumetric water content, mosquito abundance | Y | N | N | N | Land usage impacts | N | (38) |
|  | Yamana (2013) | Investigate effect of acquired immunity on malaria prevalence in varying locations | H,M | N | N | Y | P,C,V,S | Spatial distribution of water depths and temperatures | Malaria prevalence | Y | N | Y | N | Acquired immunity | N | (39) |
|  | Bomblies (2014) | Assess impact of larvicide use and pool drainage on vector populations | H,M | N | Y | Y | P,C,V,S | Precipitation, temperature, humidity, radiation, wind speed and direction | Water pool vectorial capacity, mosquito population | Y | N | N | N | CO_2_ gradients | N | (40) |
|  | Yamana (2016) | Demonstration concept of hysteresis in malaria transmission | H,M | N | N | Y | P,C,V | Entomological parameters, initial malaria prevalence | Malaria prevalence, immunity | Y | N | Y | N | Impact of 11 years of climate data on later years | N | (41) |
|  | Endo (2016) | Provide tool to analyse malaria transmission potential under varying environmental situations | H,M | N | N | Y | P,C,S | House location, rainfall and groundwater parameters | Basic reproductive rate, length of wet season required for stable malaria transmission | Y | N | Y | N | Analysing impact of wet season length on transmission | N | (42) |
|  | Endo (2018) | Investigate environmental impact on malaria transmission around reservoirs | H,M | N | N | Y | P | Meteorological data, water levels, annual incidence rate | Relative malaria infection, *Anopheles* population | Y | N | N | N | Decomposition of the impact of environmental factors | N | (43) |
| Filipe (2007) | |  |  |  |  |  |  |  |  |  |  |  |  |  |  | (44) |
|  | Griffin (2010) | Assess changing impact of past, current and future intervention strategies in SSA | H | Y | Y | N | P,C,S | EIR, intervention coverage, seasonality parameters | Parasite prevalence, reduction in parasite prevalence | N | Y | Y | N | Dynamic host immunity, superinfection, liver stages of parasite | N | (45) |
|  | Okell (2011) | Explore short- and long-term impacts of MDA in various transmission scenarios | H | Y | Y | N | P,C,V,S | Pregnancy prevalence, drug and parasite parameters | EIR, prevalence of infection | N | Y | Y | N | Pregnancy prevalence | N | (46) |
|  | Griffin (2014) | Estimate how the age-burden of *P. falciparum* malaria is changing in SSA | H | Y | N | N | P,C,V,S | Current malaria prevalence in 2-10 year olds | Age-stratified proportion of cases, yearly incidence | N | Y | Y | N | Immunity functions | N | (47) |
|  | Okell (2014) | CEA of first-line malaria treatments | H | Y | Y | N | P,C,V, S | Cost input data, local transmission parameters | Cost difference between treatments, reduction in cases | N | Y | Y | Y | PK, PD, access to treatment, health system costs | N | (48) |
|  | Slater (2014) | Assess impact of adding ivermectin to a mass treatment intervention on malaria transmission | H | N | Y | N | P, C | Drug properties, malaria test properties | Time to interrupt transmission, RDT-positivity, vector mortality outcomes | N | N | Y | N | Variably IVM impact considering time between dosing and vector blood meal | N | (49) |
|  | Griffin (2015) | Model acquisition and loss of immunity to age distribution of severe malaria cases | H | Y | N | N | C,V | EIR, incidence by age group | Probability of severe malaria, proportion with specific symptoms, case fatality percentage | N | Y | Y | N | Differing manifestations of severe malaria, immunity functions | N | (50) |
|  | Cairns (2015) | Determine when long-acting ACT is most beneficial | H | Y | Y | Y | P,C,S | Transmission and seasonality parameters | Malaria burden, seasonality of incidence | N | N | Y | N | Markham Seasonality Index | N | (51) |
|  | Slater (2016) | Simulate impact of artemisinin and partner drug resistance in SSA | H | Y | Y | N | P,C,S | Parasite clearance rates, recrudescence rates, seasonality parameters | Additional cases due to resistance | N | N | Y | N | Drug resistance | N | (52) |
|  | Griffin (2016) | Assess impact of future intervention scenarios on malaria transmission | H | Y | Y | N | P | Pre-existing malaria rates and intervention use | Cases and deaths averted, at-risk population | N | Y | Y | N | Existing intervention coverage, insecticide decay, ITN wear-and-tear | N | (53) |
|  | Walker (2016) | Estimate the minimum-cost ordering of interventions to reduce malaria burden and transmission | H | Y | Y | N | P,S | Cost price of interventions, interventions efficacies and coverages, transmission intensity, seasonality, vector profiles | Cost of disease burden or transmission reduction | N | N | Y | Y | Simulated annealing for optimisation | Y | (54) |
|  | Winskill (2016) | Compare the cost-effectiveness of RTS,S vaccination with increasing pre-existing interventions in SSA | H | Y | Y | N | S | Baseline parasite prevalence, seasonal transmission patterns, vector profiles, intervention choice and coverage level | Cases averted, DALYs averted, costs | N | Y | Y | Y | Method for cost-effective scale-up of intervnetions | Y | (55) |
|  | Bretscher (2017) | Impact of antimalarial type and population delivery modality on transmission | H | Y | Y | N | P,C,S | Infection and transmission parameters, drug efficacies, intervention coverage and efficacy | Reduction in clinical cases | N | N | Y | N |  | N | (56) |
|  | Watson (2017) | Impact of rapid diagnostic tests (RDTs) on PfHRP2-geleted parasites | H | Y | Y | N | P,C,S | Infection, treatment and transmission probabilities | Rate at which population infected only with PfHRP2-deleted mutants | N | N | Y | N | PfHRP2 dynamics, immunity | N | (57) |
| Churcher (2010) | | Assess the impact of vector parasite densities on transmission-blocking interventions (TBIs) | M | Y | Y | N | P | Vector parasite density parameters, HBR | Prevalence of infectious mosquitoes, density of salivary gland sporozoites | N | N | N | N | Mosquito parasite densities | N | (58) |
| Gatton (2004) | |  |  |  |  |  |  |  |  |  |  |  |  |  |  | (59) |
|  | Gatton (2010) | Compare interventions in low to moderate transmission settings | H, | Y | Y | Y | P,V,S | EIR, treatment coverage, bednet coverage, parasite and mosquito-related parameters | Interruption of transmission | N | N | Y | N | Genetic variation of *P. falciparum*, liver stage parasite development | N | (60) |
|  | Gatton (2017) | Investigate impact of PfHRP2 presence in parasite populations on malaria-based outcomes | H | Y | Y | N | P | Parasite PfHRP status, rapid diagnostic test (RDT) type, vector survival characteristics | EIR, time to extinction for parasite types, RDT positivity %, number of infections by parasite type | N | N | Y | N | Pharmacokinetics, parasite PfHRP2 status | N | (61) |
| Zhou (2010) | |  |  |  |  |  |  |  |  |  |  |  |  |  |  | (62) |
|  | Arifin (2011) | Compare output of spatial and non-spatial ABM, investigate impact of environmental heterogeneity on vector abundance | M | N | N | Y | V,S | Aquatic habitat density, bloodmeal density | Vector abundance | Y | N | N | N | Potential for age-specific mortality rates for vectors. | N | (63) |
|  | Arifin (2013) | Compare results with Gu (2009A, B), assess ITN and LSM on vector populations | H,M | N | Y | N | Nil | Spatial landscape, intervention efficacy parameters | Female adult mosquito abundance | Y | N | N | N | "Docking" technique of cross-validation | N | (64) |
|  | Arifin (2014) | Compare impact of hypothetical vector control interventions (HVCIs) on vector prevalence | M | Y | Y | Y | Nil | Coverage and killing capacity of interventions, environmental descriptors | Female adult mosquito abundance, potentially infectious mosquito abundance | N | N | N | N | Hypothetical interventions that can reflect real strategies | N | (65) |
|  | Gentile (2015) | Assess impact of sterile insect technique (SIT) on female *A. gambiae* population | M | N | Y | Y | S | Mosquito state transition parameters | Female wildtype proportion | N | N | N | N | Mating competitiveness of transgenic male vectors | N | (66) |
|  | Arifin (2015) | Present a framework that integrates ABM outputs with a geographic information system (GIS) | M | N | Y | Y | Nil | Intervention coverage | Mosquito abundance, oviposition counts per habitat, blood meal count per house | Y | N | N | N | Hot spot analysis to identify significant locations, use of GIS with ABM | N | (67) |
|  | Alam (2017) | Adapt *A. gambiae* ABM to *A. vagus*, assess interventions in Bandarban, Bangladesh | M | N | Y | Y | P,V | Daily temperature and rainfall, vector active times, landscape information | Female abundance, abundance of vector life stages | Y | N | N | N | Docking validation technique, temperature-dependant vector development | N | (68) |
| Eckhoff (2011) | | Outline new model, assess impact of various interventions on malaria transmission | H,M | N | Y | Y | P,C,S | Probabilities of successful actions | HBR, EIR, adult mortality | N | N | N | N | Animal blood meals, indoor feeding, preferential and variable larval survival | N | (69) |
|  | Eckhoff (2012) | Investigate the acquisition of immunity and its dependence on age and exposure | H | Y | N | N | P | Parasite characteristics immunity and development parameters, rainfall, temperature | Malaria prevalence, mean parasitaemia | N | N | Y | N | PfEMP-1 surface antigen variation and highly specific parasite development | N | (70) |
|  | Eckhoff (2013) | Model effects of multiple interventions on malaria transmission | H,M | N | Y | Y | P,C,V,S | Intervention coverage and efficacy, EIR, vector endophagy | Prevalence, daily EIR | N | N | N | N | Multiple vector species modelled | N | (71) |
|  | Wenger (2013) | Model expected impacts of present and future vaccines | H | Y | Y | Y | P,S | Climate and vector parameters, EIR | Prevalence, daily EIR, daily clinical incidence rate | N | N | Y | N |  | N | (72) |
|  | McCarthy (2015) | Calibrate Eckhoff (2011) to immune system parameters, evaluate impact of pre-erythrocytic vaccine in varying transmission conditions | H,M | Y | Y | Y | P,C,V | Age-stratified prevalence, fever incidence, age-stratified severe disease incidence | Parasite prevalence, severe disease incidence, per-bite vaccine efficacy | N | Y | Y | N | Severe malaria diagnosis, parasite antigenic variation, innate and adaptive immunity | N | (73) |
|  | Gerardin (2015A) | Comparison of antimalarials using pharmacokinetic and pharmacodynamic modelling | H,M | N | Y | Y | P,C,S | Environmental data, EIR, parameters relating to antimalarial pharmacokinetics | Asexual parasite prevalence | N | N | Y | N | Pharmacokinetics (PK), pharmacodynamics (PD) | N | (74) |
|  | Gerardin (2015B) | Characterise infectious reservoir by age and detection threshold, without intervention and under targeted elimination campaigns | H,M | Y | Y | Y | P,C | Age-stratified prevalence and clinical incidence data, EIRs, age- and season-stratified asexual parasite and gametocyte densities | Asexual parasite and gametocyte densities | N | Y | Y | N | Importation of cases | N | (75) |
|  | Gerardin (2016) | Estimate household exposure to malaria transmission based on spatial infection status in Zambia | H | N | Y | Y | C | Household location, age structure, RDT positivity by age (for population structure) | Transmission intensity per household, number of clinical cases and new infections averted, fraction of infected individuals treated, probability of less than one onward infection | Y | N | Y | N | Importation of cases | Y | (76) |
|  | Eckhoff (2016) | Simulate the impacts of gene-drive mosquitoes on mosquito populations in SSA environments | M | N | Y | Y | P,S | Homing rate, fecundity reduction | Wildtype fraction, adult population size, EIR reduction | Y | N | N | N |  | N | (77) |
|  | Gerardin (2017) | Evaluate the effects of reactive case detection on malaria transmission | H,M | Y | Y | Y | P,C | Baseline transmission intensity | Malaria prevalence, incidence, fraction of simulations with no local transmission | Y | N | Y | N | *Anopheles*-specific biting habits, best interventions package differing between transmission settings | N | (78) |
|  | Gerardin (2018) | Examine effect of MDA in villages on transmission in surrounding area | H | Y | Y | N | Nil | Biting rates, travel timing, intervention timing | Percentage reduction in prevalence one year post-MDA administration | N | N | Y | N | Demographic cycling, human migration | Y | (79) |
|  | Ouédraogo (2018) | Impact of parasite sexual-stage immunity on human infectious reservoir | H | Y | N | N | P,V | Malaria incidence, sexual-stage antibody concentrations | Frequency of oocyst density per mosquito, percentage of infected mosquitoes | N | N | Y | N | Boost and decay of sexual-stage antibodies | N | (80) |
| Klein (2014) | | Explore impact of heterogeneous transmission on population spread of drug resistance | H,M | Y | N | N | P | Various human, mosquito and transmission parameters, EIR | Population disease states, multiplicity of infection (MOI), time to resistance | N | N | N | N | Parasite clones, drug resistance | N | (81) |
| Zhu (2015A) | | Assess effects of location and density of sugar sources and resting sites on vector epidemiology | H,M | N | N | Y | P | Sugar source location and frequency | Mean daily survival rate, mean human biting rate, mean daily abundance | Y | N | N | N | Location of sugar sources, specific human movements | N | (82) |
|  | Zhu (2015B) | Assess effect of attractive toxic sugar baits (ATSB) on vector and malaria epidemiology | H,M | N | Y | Y | P | ATSB location and frequency | EIR, mean HBR, mean daily abundance | Y | N | N | N | ATSB, specific human movements | Y | (83) |
|  | Zhu (2017) | Assess ATSB and LLIN use in combination for vector control and malaria transmission impacts | H,M | N | Y | Y | P | ATSB location and frequency, LLIN coverage levels | EIR, vector populations, probability of local vector extinction | Y | N | N | N | Integrated vector management (IVM), specific human movements | N | (84) |
| Phillips (2015) | | Assess cost-effectiveness of diagnostic testing of malaria in children with fever | H | N | Y | N | P,C,S | Fever transmission rates, clinician compliance rates, intervention costs | Total costs, deaths averted | N | N | N | Y | Patient, carer and clinician behaviour | N | (85) |
| Silal (2015A) | |  |  |  |  |  |  |  |  |  |  |  |  |  |  | (86) |
|  | Silal (2015B) | Assess impact of border-control focal screen-and-treat campaign on transmission | H | N | Y | N | P,C,V | Parameters of infection and human behaviour for compartment flow | Reduction in local infections | Y | N | N | N | Focus on border-control, ABM contained within larger deterministic model | N | (87) |
| Nguyen (2015) | | Compare strategies for optimum use of ACTs at the population level, without accelerating drug resistance | H | Y | Y | N | P,C,V,S | Cost of resistance, treatment coverage | Treatment failure rate, useful therapeutic life, resistance emergence, partner drug resistance rates | N | N | Y | N | Drug resistance, PK/PD | Y | (88) |
| Pizzitutti (2015) | | Represent changes in malaria transmission with Amazonian river levels near Padre Cocha, demonstrate effectiveness of larval habitat control | H,M | Y | Y | Y | C,V | River level, larval habitat control buffer radius | Malaria incidence, spatial clustering of cases | Y | N | N | N | Parasites as agents, risk-dependent implementation of interventions | N | (89) |
|  | Pizzitutti (2018) | Assess influence of human movement on local-scale malaria transmission | H,M | Y | Y | Y | P,C,V | River level, meteorological data, human movement characteristics | Malaria incidence | Y | N | Y | N | Age- and job-specific movement patterns, asymptomatic hosts | N | (90) |
| Sauboin (2015) | | Assess candidate malaria vaccine in SSA transmission environments | H | N | Y | N | P,C,V,S | Transition probabilities, CFR, vaccine efficacy | Reduction in cases, severe cases, hospitalisations, deaths | N | N | Y | N | Full immunity | N | (91) |
| Macdonald (1952) | |  |  |  |  |  |  |  |  |  |  |  |  |  |  | (92) |
|  | Karl (2016) | Assess usefulness of spatially heterogeneous ABM to determine MOI and EIR in PNG | H,M | Y | N | Y | P,C,S | Transmission rate, mosquito density, human-to-mosquito ratio, MOI, prevalence | EIR, MOI, malaria prevalence | Y | N | Y | N | Multiclonal infection, based on AV in PNG, *P. falciparum* clone distribution | N | (93) |
| Ferreira (2017) | | Assess changes to mosquito biting behaviour due to long term ITN use | M | N | Y | N | P,S | Vector biting time, ITN use and effectiveness, variance of parent/child parameters | Vector mortality probability, oviposition probability, timing of blood meals | N | N | N | N | Genetic plasticity of mosquitoes, vector-specific biting times | N | (94) |
| Choi (2017) | | Assess cost-effectiveness of antimalarial treatments for HIV-positive pregnant women | H | N | Y | N | P,S | Costing parameters, intervention efficacy parameters, country-level disease parameters | Reductions in low birth weight, anaemia, malaria parasitaemia | N | N | N | Y | Human agents all HIV-positive pregnant women | N | (95) |
| Shcherbacheva (2017A) | | Assess impacts of LLINs on host-seeking behaviour of mosquitoes | M | N | Y | N | P,C | Intervention effect parameters, vector movement parameters | Death rate, hut exit rate, vector feeding rate, R_0_ | Y | N | N | N | CO_2_ gradient, simulation of individual hut, vector consumption of chemical | N | (96) |
|  | S’cheva (2017B) | Study alterations in vector behaviour due to parasites and household size | H,M | N | N | N | P,C | Household size, biting rates | R_0_, contact rate | Y | N | N | N | CO_2_ gradient, enhanced attractiveness of hosts for vectors, change in biting rates for infected vectors | N | (97) |

## References

1. McKenzie FE, Wong RC, Bossert WH. Discrete-Event Simulation Models of Plasmodium falciparum Malaria. Simulation [Internet]. 1998;71(4):250–61. Available from: http://www.pubmedcentral.nih.gov/articlerender.fcgi?artid=2490820&tool=pmcentrez&rendertype=abstract

2. Mckenzie FE, Killeen GF, Beier JC, Bossert WH. Seasonality , Parasite Diversity , and Local Extinctions in Plasmodium falciparum Malaria. 2001;82(10):2673–81.

3. McKenzie FE, Bossert WH. An integrated model of Plasmodium falciparum dynamics. J Theor Biol. 2005;232(3):411–26.

4. Gu W, Killeen GF, Mbogo CM, Regens JL, Githure JI, Beier JC. An individual-based model of Plasmodium falciparum malaria transmission on the coast of Kenya. Trans R Soc Trop Med Hygeine. 2003;97(1):43–50.

5. Gu W, Novak RJ. Agent-based modelling of mosquito foraging behaviour for malaria control. Trans R Soc Trop Med Hygeine. 2009;103(11):1105–18.

6. Gu W, Novak RJ. Predicting the impact of insecticide-treated bed nets on malaria transmission: the devil is in the detail. Malar J. 2009;8(1):256–65.

7. Depinay J-MO, Mbogo CM, Killeen G, Knols B, Beier J, Carlson J, et al. A simulation model of African Anopheles ecology and population dynamics for the analysis of malaria transmission. Malar J. 2004;3(1):29–49.

8. Molineaux L, Diebner HH, Eichner M, Collins WE, Jeffery GM, Dietz K. Plasmodium falciparum parasitaemia described by a new mathematical model. Parasitology. 2001;122(Pt 4):379–91.

9. Dietz K, Raddatz G, Molineaux L. Mathematical model of the first wave of Plasmodium falciparum asexual parasitemia in non-immune and vaccinated individuals. Am J Trop Med Hyg. 2006;75(2 suppl):46–55.

10. Rateb F, Pavard B, Bellamine-BenSaoud N, Merelo JJ, Arenas MG. Modeling Malaria with Multi-Agent Systems. Int J Intell Inf Technol. 2005;1(June):17–27.

11. Smith T, Killeen GF, Maire N, Ross A, Molineaux L, Tediosi F, et al. Mathematical modeling of the impact of malaria vaccines on the clinical epidemiology and natural history of Plasmodium falciparum malaria: Overview. Am J Trop Med Hyg. 2006;75(2 suppl):1–10.

12. Smith T, Maire N, Dietz K, Killeen GF, Vounatsou P, Molineaux L, et al. Relationship between the entomologic inoculation rate and the force of infection for Plasmodium falciparum malaria. Am J Trop Med Hyg. 2006;75(2 suppl):11–8.

13. Maire N, Smith T, Ross A, Owusu-Agyei S, Dietz K, Molineaux L. A model for natural immunity to asexual blood stages of Plasmodium falciparum malaria in endemic areas. Am J Trop Med Hyg. 2006;75(2 Suppl):19–31.

14. Ross A, Killeen G, Smith T. Relationships between host infectivity to mosquitoes and asexual parasite density in Plasmodium falciparum. Am J Trop Med Hyg. 2006;75(2 suppl):32–7.

15. Killeen GF, Ross A, Smith T. Infectiousness of malaria-endemic human populations to vectors. Am J Trop Med Hyg. 2006;75(2 Suppl):38–45.

16. Smith T, Ross A, Maire N, Rogier C, Trape J-F, Molineaux L. An epidemiologic model of the incidence of acute illness in Plasmodium falciparum malaria. Am J Trop Med Hyg. 2006;75(2 suppl):56–62.

17. Ross A, Maire N, Molineaux L, Smith T. An epidemiologic model of severe morbidity and mortality caused by Plasmodium falciparum. Am J Trop Med Hyg. 2006;75(2 suppl):63–73.

18. Tediosi F, Maire N, Smith T, Hutton G, Utzinger J, Ross A, et al. An approach to model the costs and effects of case management of Plasmodium falciparum malaria in sub-saharan Africa. Am J Trop Med Hyg. 2006;75(2 Suppl):90–103.

19. Maire N, Aponte JJ, Ross A, Thompson R, Alonso P, Utzinger J, et al. Modeling A Field Trial of the RTS,S/AS02A Malaria Vaccine. Am J Trop Med Hyg. 2006;75(2 suppl):104–10.

20. Maire N, Tediosi F, Ross A, Smith T. Predictions of the epidemiologic impact of introducing a pre-erythrocytic vaccine into the expanded program on immunization in sub-Saharan Africa. Am J Trop Med Hyg. 2006;75(2 suppl):111–8.

21. Tediosi F, Hutton G, Maire N, Smith TA, Ross A, Tanner M. Predicting the Cost-Effectiveness of Introducing a Pre-Erythrocytic Malaria Vaccine Into the Expanded Program on Immunization in Tanzania. Am J Trop Med Hyg. 2006;75(2 suppl):119–30.

22. Smith T, Maire N, Ross A, Penny M, Chitnis N, Schapira A, et al. Towards a comprehensive simulation model of malaria epidemiology and control. Parasitology. 2008;135(13):1507–16.

23. Ross A, Penny M, Maire N, Studer A, Carneiro I, Schellenberg D, et al. Modelling the epidemiological impact of intermittent preventive treatment against malaria in infants. PLoS One. 2008;3(7):e2661.

24. Penny MA, Maire N, Studer A, Schapira A, Smith TA. What should vaccine developers ask? Simulation of the effectiveness of malaria vaccines. PLoS One. 2008;3(9).

25. Tediosi F, Maire N, Penny M, Studer A, Smith TA. Simulation of the cost-effectiveness of malaria vaccines. Malar J. 2009;8(1):127–43.

26. Ross A, Smith T. Interpreting malaria age-prevalence and incidence curves: a simulation study of the effects of different types of heterogeneity. Malar J. 2010;9(1):132–40.

27. Ross A, Maire N, Sicuri E, Smith T, Conteh L. Determinants of the cost-effectiveness of intermittent preventive treatment for malaria in infants and children. PLoS One. 2011;6(4):e18391.

28. Maire N, Shillcutt SD, Walker DG, Tediosi F, Smith TA. Cost-effectiveness of the introduction of a pre-erythrocytic malaria vaccine into the expanded program on immunization in sub-Saharan Africa: Analysis of uncertainties using a stochastic individual-based simulation model of Plasmodium falciparum malaria. Value Heal. 2011;14(8):1028–38.

29. Chitnis N, Hardy D, Smith T. A Periodically-Forced Mathematical Model for the Seasonal Dynamics of Malaria in Mosquitoes. Bull Math Biol. 2012;74(5):1098–124.

30. Crowell V, Briët OJ, Hardy D, Chitnis N, Maire N, Pasquale A Di, et al. Modelling the cost-effectiveness of mass screening and treatment for reducing Plasmodium falciparum malaria burden. Malar J [Internet]. 2013 Jan 3 [cited 2018 Jun 4];12(1):4. Available from: http://malariajournal.biomedcentral.com/articles/10.1186/1475-2875-12-4

31. Richard A, Richardson S, Maccario J. A three-state Markov model of Plasmodium falciparum parasitemia. Math Biosci. 1993 Sep 1;117(1–2):283–300.

32. Gurarie D, McKenzie FE. A stochastic model of immune-modulated malaria infection and disease in children. Math Biosci. 2007;210(2):576–97.

33. Gurarie D, Karl S, Zimmerman PA, King CH, St Pierre TG, Davis TME. Mathematical modeling of malaria infection with innate and adaptive immunity in individuals and agent-based communities. PLoS One. 2012;7(3):e34040.

34. Linard C, Ponçon N, Fontenille D, Lambin EF. A multi-agent simulation to assess the risk of malaria re-emergence in southern France. Ecol Modell. 2009;220(2):160–74.

35. Bomblies A, Duchemin JB, Eltahir EAB. Hydrology of malaria: Model development and application to a Sahelian village. Water Resour Res. 2008;44(12):1–26.

36. Bomblies A, Duchemin J-B, Eltahir EAB. A mechanistic approach for accurate simulation of village scale malaria transmission. Malar J. 2009;8(1):223–34.

37. Bomblies A, Eltahir EAB. Assessment of the impact of climate shifts on malaria transmission in the Sahel. Ecohealth. 2009;6(3):426–37.

38. Stryker JJ, Bomblies A. The impacts of land use change on malaria vector abundance in a water-limited, highland region of Ethiopia. Ecohealth. 2012;9(4):455–70.

39. Yamana TK, Bomblies A, Laminou IM, Duchemin J-B, Eltahir EAB. Linking environmental variability to village-scale malaria transmission using a simple immunity model. Parasit Vectors [Internet]. 2013;6(1):226–39. Available from: http://dx.doi.org/10.1186/1756-3305-6-226

40. Bomblies A. Agent-based modeling of malaria vectors: the importance of spatial simulation. Parasit Vectors. 2014;7(1):308–17.

41. Yamana TK, Qiu X, Eltahir EAB. Hysteresis in simulations of malaria transmission. Adv Water Resour [Internet]. 2017;108:416–22. Available from: http://dx.doi.org/10.1016/j.advwatres.2016.10.003

42. Endo N, Eltahir EAB. Environmental determinants of malaria transmission in African villages. Malar J. 2016;15(1):1–11.

43. Endo N, Eltahir EAB. Environmental Determinants of Malaria Transmission Around the Koka Reservoir in Ethiopia. GeoHealth [Internet]. 2018;2(3):104–15. Available from: http://doi.wiley.com/10.1002/2017GH000108

44. Filipe JAN, Riley EM, Drakeley CJ, Sutherland CJ, Ghani AC. Determination of the processes driving the acquisition of immunity to malaria using a mathematical transmission model. PLoS Comput Biol. 2007;3(12):2569–79.

45. Griffin JT, Hollingsworth TD, Okell LC, Churcher TS, White M, Hinsley W, et al. Reducing Plasmodium falciparum malaria transmission in Africa: a model-based evaluation of intervention strategies. PLoS Med. 2010;7(8):e1000324.

46. Okell LC, Griffin JT, Kleinschmidt I, Hollingsworth TD, Churcher TS, White MJ, et al. The potential contribution of mass treatment to the control of plasmodium falciparum malaria. PLoS One. 2011;6(5).

47. Griffin JT, Ferguson NM, Ghani AC. Estimates of the changing age-burden of Plasmodium falciparum malaria disease in sub-Saharan Africa. Nat Commun. 2014;5:1–10.

48. Okell LC, Cairns M, Griffin JT, Ferguson NM, Tarning J, Jagoe G, et al. Contrasting benefits of different artemisinin combination therapies as first-line malaria treatments using model-based cost-effectiveness analysis. Nat Commun [Internet]. 2014 Dec 26 [cited 2018 Jun 4];5(1):5606. Available from: http://www.nature.com/articles/ncomms6606

49. Slater HC, Walker PGT, Bousema T, Okell LC, Ghani AC. The potential impact of adding ivermectin to a mass treatment intervention to reduce malaria transmission: A modelling study. J Infect Dis. 2014;210(12):1972–80.

50. Griffin JT, Hollingsworth TD, Reyburn H, Drakeley CJ, Riley EM, Ghani AC. Gradual acquisition of immunity to severe malaria with increasing exposure. Proc R Soc B Biol Sci [Internet]. 2015 Feb 22 [cited 2018 Jun 4];282(1801):20142657–20142657. Available from: http://www.ncbi.nlm.nih.gov/pubmed/25567652

51. Cairns ME, Walker PGT, Okell LC, Griffin JT, Garske T, Asante KP, et al. Seasonality in malaria transmission: implications for case-management with long-acting artemisinin combination therapy in sub-Saharan Africa. Malar J. 2015;14(1):321–33.

52. Slater HC, Griffin JT, Ghani AC, Okell LC. Assessing the potential impact of artemisinin and partner drug resistance in sub-Saharan Africa. Malar J. 2016;15(1):10–20.

53. Griffin JT, Bhatt S, Sinka ME, Gething PW, Lynch M, Patouillard E, et al. Potential for reduction of burden and local elimination of malaria by reducing Plasmodium falciparum malaria transmission: A mathematical modelling study. Lancet Infect Dis [Internet]. 2016 Apr 1 [cited 2018 Jun 4];16(4):465–72. Available from: https://www.sciencedirect.com/science/article/pii/S1473309915004235#bib6

54. Walker PGT, Griffin JT, Ferguson NM, Ghani AC. Estimating the most efficient allocation of interventions to achieve reductions in Plasmodium falciparum malaria burden and transmission in Africa: A modelling study. Lancet Glob Heal [Internet]. 2016 Jul 1 [cited 2018 Jun 4];4(7):e474–84. Available from: https://www.sciencedirect.com/science/article/pii/S2214109X16300730

55. Winskill P, Walker PG, Griffin JT, Ghani AC. Modelling the cost-effectiveness of introducing the RTS,S malaria vaccine relative to scaling up other malaria interventions in sub-Saharan Africa. BMJ Glob Heal [Internet]. 2017 [cited 2018 Jun 4];2(1):e000090. Available from: http://gh.bmj.com/content/bmjgh/2/1/e000090.full.pdf

56. Bretscher MT, Griffin JT, Ghani AC, Okell LC. Modelling the benefits of long-acting or transmission-blocking drugs for reducing Plasmodium falciparum transmission by case management or by mass treatment. Malar J. 2017;16(1):1–12.

57. Watson OJ, Slater HC, Verity R, Parr JB, Mwandagalirwa MK, Tshefu A, et al. Modelling the drivers of the spread of Plasmodium falciparum hrp2 gene deletions in sub-Saharan Africa. Elife [Internet]. 2017;6:e25008. Available from: http://elifesciences.org/lookup/doi/10.7554/eLife.25008

58. Churcher TS, Dawes EJ, Sinden RE, Christophides GK, Koella JC, Basáñez M-G. Population biology of malaria within the mosquito: density-dependent processes and potential implications for transmission-blocking interventions. Malar J. 2010;9(1):311.

59. Gatton ML, Cheng Q. Investigating antigenic variation and other parasite–host interactions in Plasmodium falciparum infections in naïve hosts. Parasitology. 2004;128(4):367–76.

60. Gatton ML, Cheng Q. Interrupting malaria transmission: Quantifying the impact of interventions in regions of low to moderate transmission. PLoS One. 2010;5(12):e15149.

61. Gatton ML, Dunn J, Chaudhry A, Ciketic S, Cunningham J, Cheng Q. Implications of parasites lacking plasmodium falciparum histidine-rich protein 2 on Malaria morbidity and control when rapid diagnostic tests are used for diagnosis. J Infect Dis [Internet]. 2017 Apr 1 [cited 2018 May 22];215(7):1156–66. Available from: https://academic.oup.com/jid/article-lookup/doi/10.1093/infdis/jix094

62. Zhou Y, Arifin SM, Gentile J, Kurtz SJ, Davis GJ, Wendelberger BA. An agent-based model of the Anopheles gambiae mosquito life cycle. Proc 2010 Summer Comput Simul Conf. 2010;1(1):201–8.

63. Arifin SMN, Davis GJ, Zhou Y. A Spatial Agent-Based Model of Malaria: Model Verification and Effects of Spatial Heterogeneity. Int J Agent Technol Syst [Internet]. 2011;3(3):17–34. Available from: http://services.igi-global.com/resolvedoi/resolve.aspx?doi=10.4018/jats.2011070102

64. Arifin SMN, Madey GR, Collins FH. Examining the impact of larval source management and insecticide-treated nets using a spatial agent-based model of Anopheles gambiae and a landscape generator tool. Malar J. 2013;12(1):290–313.

65. Arifin SMN, Zhou Y, Davis GJ, Gentile JE, Madey GR, Collins FH. An agent-based model of the population dynamics of Anopheles gambiae. Malar J. 2014;13(1):424–42.

66. Gentile JE, Rund SSC, Madey GR. Modelling sterile insect technique to control the population of Anopheles gambiae. Malar J. 2015;14(1):92–103.

67. Arifin S, Arifin R, Pitts D, Rahman M, Nowreen S, Madey G, et al. Landscape Epidemiology Modeling Using an Agent-Based Model and a Geographic Information System. Land [Internet]. 2015 [cited 2018 Jun 4];4(2):378–412. Available from: http://www.mdpi.com/2073-445X/4/2/378/

68. Alam MSMZ, Niaz Arifin SM, Al-Amin HM, Alam MSMZ, Rahman MS. A spatial agent-based model of Anopheles vagus for malaria epidemiology: Examining the impact of vector control interventions. Malar J [Internet]. 2017 Dec 27 [cited 2018 May 23];16(1):1–20. Available from: http://malariajournal.biomedcentral.com/articles/10.1186/s12936-017-2075-6

69. Eckhoff PA. A malaria transmission-directed model of mosquito life cycle and ecology. Malar J. 2011;10(1):303–19.

70. Eckhoff P a. Malaria parasite diversity and transmission intensity affect development of parasitological immunity in a mathematical model. Malar J. 2012;11(1):419.

71. Eckhoff P. Mathematical models of within-host and transmission dynamics to determine effects of malaria interventions in a variety of transmission settings. Am J Trop Med Hyg. 2013;88(5):817–27.

72. Wenger EA, Eckhoff PA. A mathematical model of the impact of present and future malaria vaccines. Malar J. 2013;12:126.

73. McCarthy KA, Wenger EA, Huynh GH, Eckhoff PA. Calibration of an intrahost malaria model and parameter ensemble evaluation of a pre-erythrocytic vaccine. Malar J [Internet]. 2015;14(1):6. Available from: http://www.malariajournal.com/content/14/1/6

74. Gerardin J, Eckhoff P, Wenger EA. Mass campaigns with antimalarial drugs: a modelling comparison of artemether-lumefantrine and DHA-piperaquine with and without primaquine as tools for malaria control and elimination. BMC Infect Dis. 2015;15(1):144–57.

75. Gerardin J, Ouédraogo AL, McCarthy KA, Eckhoff PA, Wenger EA. Characterization of the infectious reservoir of malaria with an agent-based model calibrated to age-stratified parasite densities and infectiousness. Malar J. 2015;14(1):231–43.

76. Gerardin J, Bever CA, Hamainza B, Miller JM, Eckhoff PA, Wenger EA. Optimal Population-Level Infection Detection Strategies for Malaria Control and Elimination in a Spatial Model of Malaria Transmission. PLOS Comput Biol. 2016 Jan 14;12(1):e1004707.

77. Eckhoff PA, Wenger EA, Godfray HCJ, Burt A. Impact of mosquito gene drive on malaria elimination in a computational model with explicit spatial and temporal dynamics. Proc Natl Acad Sci U S A. 2016;114(2):E255–64.

78. Gerardin J, Bever CA, Bridenbecker D, Hamainza B, Silumbe K, Miller JM, et al. Effectiveness of reactive case detection for malaria elimination in three archetypical transmission settings: A modelling study. Malar J. 2017;16(1):1–17.

79. Gerardin J, Bertozzi-Villa A, Eckhoff PA, Wenger EA. Impact of mass drug administration campaigns depends on interaction with seasonal human movement. Int Heal [Internet]. 2018;(June):1–6. Available from: https://www.ncbi.nlm.nih.gov/pubmed/29635471

80. Ouédraogo AL, Eckhoff PA, Luty AJF, Roeffen W, Sauerwein RW, Bousema T, et al. Modeling the impact of Plasmodium falciparum sexual stage immunity on the composition and dynamics of the human infectious reservoir for malaria in natural settings. Kazura JW, editor. PLOS Pathog [Internet]. 2018 May 9;14(5):e1007034. Available from: http://dx.plos.org/10.1371/journal.ppat.1007034

81. Klein EY. The impact of heterogeneous transmission on the establishment and spread of antimalarial drug resistance. J Theor Biol. 2014;340:177–85.

82. Zhu L, Qualls WA, Marshall JM, Arheart KL, DeAngelis DL, McManus JW, et al. A spatial individual-based model predicting a great impact of copious sugar sources and resting sites on survival of Anopheles gambiae and malaria parasite transmission. Malar J. 2015;14(1):59–73.

83. Zhu L, Marshall JM, Qualls WA, Schlein Y, McManus JW, Arheart KL, et al. Modelling optimum use of attractive toxic sugar bait stations for effective malaria vector control in Africa. Malar J. 2015;14(1):492–503.

84. Zhu L, Müller GC, Marshall JM, Arheart KL, Qualls WA, Hlaing WM, et al. Is outdoor vector control needed for malaria elimination? An individual-based modelling study. Malar J [Internet]. 2017 Dec 3 [cited 2018 May 24];16(1):266. Available from: http://malariajournal.biomedcentral.com/articles/10.1186/s12936-017-1920-y

85. Phillips V, Njau J, Li S, Kachur P. Simulations show diagnostic testing for malaria in young African children can be cost-saving or cost-effective. Heal Aff. 2015;34(7):1196–203.

86. Silal SP, Little F, Barnes KI, White LJ. Hitting a moving target: A model for malaria elimination in the presence of population movement. PLoS One. 2015;10(12):1–16.

87. Silal SP, Little F, Barnes KI, White LJ. Predicting the impact of border control on malaria transmission: a simulated focal screen and treat campaign. Malar J [Internet]. 2015 Dec 12;14(1):268. Available from: http://www.malariajournal.com/content/14/1/268

88. Nguyen TD, Olliaro P, Dondorp AM, Baird JK, Lam HM, Farrar J, et al. Optimum population-level use of artemisinin combination therapies: A modelling study. Lancet Glob Heal. 2015;3(12):e758–66.

89. Pizzitutti F, Pan W, Barbieri A, Miranda JJ, Feingold B, Guedes GR, et al. A validated agent-based model to study the spatial and temporal heterogeneities of malaria incidence in the rainforest environment. Malar J. 2015;14(1):514–32.

90. Pizzitutti F, Pan W, Feingold B, Zaitchik B, Álvarez CA, Mena CF. Out of the net: An agent-based model to study human movements influence on local-scale malaria transmission. Kumar S, editor. PLoS One [Internet]. 2018 Mar 6 [cited 2018 May 23];13(3):e0193493. Available from: http://dx.plos.org/10.1371/journal.pone.0193493

91. Sauboin CJ, Van Bellinghen L-A, Van De Velde N, Van Vlaenderen I. Potential public health impact of RTS,S malaria candidate vaccine in sub-Saharan Africa: a modelling study. Malar J. 2015;14(1):524–40.

92. Macdonald G. The analysis of equilibrium in malaria. Trop Dis Bull. 1952 Sep;49(9):813–29.

93. Karl S, White MT, Milne GJ, Gurarie D, Hay SI, Barry AE, et al. Spatial effects on the multiplicity of Plasmodium falciparum infections. PLoS One. 2016;11(10):e0164054.

94. Ferreira CP, Lyra SP, Azevedo F, Greenhalgh D, Massad E. Modelling the impact of the long-term use of insecticide-treated bed nets on Anopheles mosquito biting time. Malar J [Internet]. 2017 Dec 15 [cited 2018 May 23];16(1):373. Available from: http://malariajournal.biomedcentral.com/articles/10.1186/s12936-017-2014-6

95. Choi SE, Brandeau ML, Bendavid E. Cost-effectiveness of malaria preventive treatment for HIV-infected pregnant women in sub-Saharan Africa. Malar J [Internet]. 2017 Dec 6 [cited 2018 May 23];16(1):1–10. Available from: http://malariajournal.biomedcentral.com/articles/10.1186/s12936-017-2047-x

96. Shcherbacheva A, Haario H, Killeen GF. Modeling host-seeking behavior of African malaria vector mosquitoes in the presence of long-lasting insecticidal nets. Math Biosci [Internet]. 2018 Jan 1 [cited 2018 May 23];295:36–47. Available from: https://www.sciencedirect.com/science/article/pii/S0025556417305473?via%3Dihub

97. Shcherbacheva A, Haario H. The Impact of Household Size on Malaria Reduction in Relation with Alterations in Mosquito Behavior by Malaria Parasite. J Multi-Valued Log Soft Comput. 2017;29:455–68.
