# Supplementary material for: Agent-based models of malaria transmission: a systematic review
Source: Malar J. 2018 Aug 17;17:299. doi: 10.1186/s12936-018-2442-y (PMC6098619; doi:10.1186/s12936-018-2442-y)
Supplement: Supplementary file 3 — Additional file 3. Adapted PRISMA search flow diagram of study selection. [file 12936_2018_2442_MOESM3_ESM.docx]

# Additional Information - Agent-Based Models of Malaria Transmission: A Systematic Review

Smith, Neal R.; Trauer, James M.; Gambhir, Manoj; Richards, Jack S.; Maude, Richard J.; Keith, Jonathan M.; Flegg, Jennifer A.

## Additional File 3: Adapted PRISMA search flow diagram of study selection.

Full-text articles assessed for eligibility: n = 137

Full-text articles excluded: n = 47

- Not malaria (n = 1)
- Not transmission modelling (n = 17)
- Not agent-based (n = 14)
- Comparison of pre-existing models (n = 15)

Records screened after duplicates removed: n = 406

Records excluded: n = 269

- Wrong study type (n = 157)
- Not malaria (n = 71)
- Not transmission modelling (n = 3)
- Not agent-based (n = 38)

Records identified through database searching:

n = 366

Additional records identified through other sources:

n = 127

Studies included in review: n = 90
