# Supplementary material for: Agent-based models of malaria transmission: a systematic review
Source: Malar J. 2018 Aug 17;17:299. doi: 10.1186/s12936-018-2442-y (PMC6098619; doi:10.1186/s12936-018-2442-y)
Supplement: Supplementary file 4 — Additional file 4. Overview of elements of spatial models. [file 12936_2018_2442_MOESM4_ESM.docx]

# Additional Information - Agent-Based Models of Malaria Transmission: A Systematic Review

Smith, Neal R.; Trauer, James M.; Gambhir, Manoj; Richards, Jack S.; Maude, Richard J.; Keith, Jonathan M.; Flegg, Jennifer A.

## Additional File 4: Overview of elements of spatial models.

### *Model does not provide explicit spatial locations, but has the capacity and structure for spatial mapping. **Grid cell size environment-dependant. †Estimated or taken from previous study. ‡No grid size provided (30 x 30 cell region). Definitions: *reflecting boundaries* - agents reflected back into study area upon reaching boundary. *Non-absorbing boundaries* – agents re-enter study on directly opposite edge upon reaching boundary. **^•^** *Location type:* R – real location, mapped out using spatial techniques. T – spatial location representing a theoretical location (e.g. a typical village structure in a defined location). H – hypothetical location bearing no specific resemblance to a physical location.

| **Model** | | **Spatial Techniques** | **Area Modelled** | **Grid/Patch Size (m)** | **Moving Agents** | **Spatial Interventions** | **Environment** | **Location type^•^** | **Reference** |
| --- | --- | --- | --- | --- | --- | --- | --- | --- | --- |
| **Original** | **Adapted** |  |  |  |  |  |  |  |  |
| Gu (2009A) | | Grid-based; absorbing boundaries | 2000 x 2000m | 50 | M | Y | Y | T | (1) |
|  | Gu (2009B) | Grid based | 2000 x 2000m | 50 | M | Y | Y | T | (2) |
| Depinay (2004) | | Simple random dispersal | N/A | N/A* | M* | Y* | Y* | H | (3) |
| Rateb (2005) | | Micro-environments | Haiti | N/A | H | Y | Y | T | (4) |
| Linard (2008) | | Grid-based | ~25km^2^ | 30 | H | Y | Y | R | (5) |
| Bomblies (2008) | | Grid-based | 2500 x 2500m | 10-80** | M | N | Y | R | (6) |
|  | Bomblies (2009A) | Banizoumbou: Grid-based | 2500 x 2500m | 10-80† | M | N | Y | R | (7) |
|  |  | Zindarou: Grid-based | 2000 x 2000m | 50 | M | N | Y | R |  |
|  | Bomblies (2009B) | Banizoumbou: Grid-based | 2500 x 2500m | 10-80† | M | N | Y | R | (8) |
|  |  | Zindarou: Grid-based | 2000 x 2000m | 50? | M | N | Y | R |  |
|  | Stryker (2012) | Grid-based | 2000 x 2000m | 10 | M | N | Y | R | (9) |
|  | Yamana (2013) | Banizoumbou: Grid-based | 2500 x 2500m | 10 | M | N | Y | R | (10) |
|  |  | Zindarou: Grid-based | 2000 x 2000m | 10 | M | N | Y | R |  |
|  | Bomblies (2014) | Grid-based | 2500 x 2500m | 10-80† | M | Y | Y | R | (11) |
|  | Yamana (2016) | Grid-based | 2500 x 2500m | 10-80 | M | Y | Y | R | (12) |
|  | Endo (2016) | Grid-based, non-absorbing boundaries | 1500 x 1000m | 10 | M | N | Y | H | (13) |
|  | Endo (2018) | Grid-based | 3000 x 3000m | 10 | M | N | Y | R | (14) |
| Zhou (2010) | |  |  |  |  |  |  |  |  |
|  | Arifin (2011) | Grid-based‡ | N/A | N/A | Y | Y | Y | H | (15) |
|  | Arifin (2013) | Grid-based, non-absorbing boundaries | 2000 x 2000m | 50 | M | Y | Y | H | (16) |
|  | Arifin (2015) | Grid-based, non-absorbing boundaries | 4800 x 4750m | 50 | M | Y | Y | H | (17) |
|  | Alam (2017) | Grid-based, non-absorbing boundaries | 2000 x 2000m | 50 | M | Y | Y | T | (18) |
| Eckhoff (2011) | |  |  |  |  |  |  |  |  |
|  | Gerardin (2016) | Patch-based | ~31.5 sq. km† | sphere radius 50 | Nil | Y | N | R | (19) |
|  | Eckhoff (2016) | Patch-based environment, grid-based movement | 10000 x 10000m | 1000 | M | Y | Y | R | (20) |
|  | Gerardin (2017) | Grid-based environment, unspecified movement | ~100km x 100km | 1000 | H,M | Y | Y | R | (21) |
| Zhu (2015A) | | Continuous landscape, reflecting boundaries | 600 x 600m | Nil | H,M | N | Y | T | (22) |
|  | Zhu (2015B) | Continuous landscape, reflecting boundaries | 600 x 600m | Nil | H,M | Y | Y | T | (23) |
|  | Zhu (2017) | Continuous landscape, reflecting boundaries | 600 x 600m | Nil | H,M | Y | Y | T | (24) |
| Silal (2015A) | |  |  |  |  |  |  |  |  |
|  | Silal (2015) | Patch-based (deterministic component) | 27850 sq. km | 1745-10250 sq.km | H | Y | N | R | (25) |
| Pizzitutti (2015) | | Grid-based | 2350 x 2520m | Geographical: 10 Mosquito: 30 | M | Y | Y | R | (26) |
|  | Pizzitutti (2018) | Padre Cocha: Grid-based | 2350 x 2520m | Geographical: 10 Mosquito: 30 | H,M | Y | Y | R | (27) |
|  |  | San Luis de Tacsha Curaray: Grid-based | 10252 x 4200m | Geographical: 10 Mosquito: 30 | H,M | Y | Y | R |  |
| Macdonald (1952) | |  |  |  |  |  |  |  |  |
|  | Karl (2016) | Distance-dependant transmission probabilities | ~35 km^2^ | N/A | H,M | N | N | R | (28) |
| Shcherbacheva (2017A) | | Patch-based | N/A | N/A | M | Y | N | H | (29) |
|  | S’cheva (2017B) | N/A | 500 x 500m | N/A | M | N | N | H | (30) |

## References

1. Gu W, Novak RJ. Agent-based modelling of mosquito foraging behaviour for malaria control. Trans R Soc Trop Med Hygeine. 2009;103(11):1105–18.

2. Gu W, Novak RJ. Predicting the impact of insecticide-treated bed nets on malaria transmission: the devil is in the detail. Malar J. 2009;8(1):256–65.

3. Depinay J-MO, Mbogo CM, Killeen G, Knols B, Beier J, Carlson J, et al. A simulation model of African Anopheles ecology and population dynamics for the analysis of malaria transmission. Malar J. 2004;3(1):29–49.

4. Rateb F, Pavard B, Bellamine-BenSaoud N, Merelo JJ, Arenas MG. Modeling Malaria with Multi-Agent Systems. Int J Intell Inf Technol. 2005;1(June):17–27.

5. Linard C, Ponçon N, Fontenille D, Lambin EF. A multi-agent simulation to assess the risk of malaria re-emergence in southern France. Ecol Modell. 2009;220(2):160–74.

6. Bomblies A, Duchemin JB, Eltahir EAB. Hydrology of malaria: Model development and application to a Sahelian village. Water Resour Res. 2008;44(12):1–26.

7. Bomblies A, Duchemin J-B, Eltahir EAB. A mechanistic approach for accurate simulation of village scale malaria transmission. Malar J. 2009;8(1):223–34.

8. Bomblies A, Eltahir EAB. Assessment of the impact of climate shifts on malaria transmission in the Sahel. Ecohealth. 2009;6(3):426–37.

9. Stryker JJ, Bomblies A. The impacts of land use change on malaria vector abundance in a water-limited, highland region of Ethiopia. Ecohealth. 2012;9(4):455–70.

10. Yamana TK, Bomblies A, Laminou IM, Duchemin J-B, Eltahir EAB. Linking environmental variability to village-scale malaria transmission using a simple immunity model. Parasit Vectors [Internet]. 2013;6(1):226–39. Available from: http://dx.doi.org/10.1186/1756-3305-6-226

11. Bomblies A. Agent-based modeling of malaria vectors: the importance of spatial simulation. Parasit Vectors. 2014;7(1):308–17.

12. Yamana TK, Qiu X, Eltahir EAB. Hysteresis in simulations of malaria transmission. Adv Water Resour [Internet]. 2017;108:416–22. Available from: http://dx.doi.org/10.1016/j.advwatres.2016.10.003

13. Endo N, Eltahir EAB. Environmental determinants of malaria transmission in African villages. Malar J. 2016;15(1):1–11.

14. Endo N, Eltahir EAB. Environmental Determinants of Malaria Transmission Around the Koka Reservoir in Ethiopia. GeoHealth [Internet]. 2018;2(3):104–15. Available from: http://doi.wiley.com/10.1002/2017GH000108

15. Arifin SMN, Davis GJ, Zhou Y. A Spatial Agent-Based Model of Malaria. Int J Agent Technol Syst [Internet]. 2011;3(3):17–34. Available from: http://services.igi-global.com/resolvedoi/resolve.aspx?doi=10.4018/jats.2011070102

16. Arifin SMN, Madey GR, Collins FH. Examining the impact of larval source management and insecticide-treated nets using a spatial agent-based model of Anopheles gambiae and a landscape generator tool. Malar J. 2013;12(1):290–313.

17. Arifin S, Arifin R, Pitts D, Rahman M, Nowreen S, Madey G, et al. Landscape Epidemiology Modeling Using an Agent-Based Model and a Geographic Information System. Land [Internet]. 2015 [cited 2018 Jun 4];4(2):378–412. Available from: http://www.mdpi.com/2073-445X/4/2/378/

18. Alam MSMZ, Niaz Arifin SM, Al-Amin HM, Alam MSMZ, Rahman MS. A spatial agent-based model of Anopheles vagus for malaria epidemiology: Examining the impact of vector control interventions. Malar J [Internet]. 2017 Dec 27 [cited 2018 May 23];16(1):1–20. Available from: http://malariajournal.biomedcentral.com/articles/10.1186/s12936-017-2075-6

19. Gerardin J, Bever CA, Hamainza B, Miller JM, Eckhoff PA, Wenger EA. Optimal Population-Level Infection Detection Strategies for Malaria Control and Elimination in a Spatial Model of Malaria Transmission. PLOS Comput Biol. 2016 Jan 14;12(1):e1004707.

20. Eckhoff PA, Wenger EA, Godfray HCJ, Burt A. Impact of mosquito gene drive on malaria elimination in a computational model with explicit spatial and temporal dynamics. Proc Natl Acad Sci U S A. 2016;114(2):E255–64.

21. Gerardin J, Bever CA, Bridenbecker D, Hamainza B, Silumbe K, Miller JM, et al. Effectiveness of reactive case detection for malaria elimination in three archetypical transmission settings: A modelling study. Malar J. 2017;16(1):1–17.

22. Zhu L, Qualls WA, Marshall JM, Arheart KL, DeAngelis DL, McManus JW, et al. A spatial individual-based model predicting a great impact of copious sugar sources and resting sites on survival of Anopheles gambiae and malaria parasite transmission. Malar J. 2015;14(1):59–73.

23. Zhu L, Marshall JM, Qualls WA, Schlein Y, McManus JW, Arheart KL, et al. Modelling optimum use of attractive toxic sugar bait stations for effective malaria vector control in Africa. Malar J. 2015;14(1):492–503.

24. Zhu L, Müller GC, Marshall JM, Arheart KL, Qualls WA, Hlaing WM, et al. Is outdoor vector control needed for malaria elimination? An individual-based modelling study. Malar J [Internet]. 2017 Dec 3 [cited 2018 May 24];16(1):266. Available from: http://malariajournal.biomedcentral.com/articles/10.1186/s12936-017-1920-y

25. Silal SP, Little F, Barnes KI, White LJ. Predicting the impact of border control on malaria transmission: a simulated focal screen and treat campaign. Malar J [Internet]. 2015 Dec 12;14(1):268. Available from: http://www.malariajournal.com/content/14/1/268

26. Pizzitutti F, Pan W, Barbieri A, Miranda JJ, Feingold B, Guedes GR, et al. A validated agent-based model to study the spatial and temporal heterogeneities of malaria incidence in the rainforest environment. Malar J. 2015;14(1):514–32.

27. Pizzitutti F, Pan W, Feingold B, Zaitchik B, Álvarez CA, Mena CF. Out of the net: An agent-based model to study human movements influence on local-scale malaria transmission. PLoS One. 2018;13(3).

28. Karl S, White MT, Milne GJ, Gurarie D, Hay SI, Barry AE, et al. Spatial effects on the multiplicity of Plasmodium falciparum infections. PLoS One. 2016;11(10):e0164054.

29. Shcherbacheva A, Haario H, Killeen GF. Modeling host-seeking behavior of African malaria vector mosquitoes in the presence of long-lasting insecticidal nets. Math Biosci [Internet]. 2018 Jan 1 [cited 2018 May 23];295:36–47. Available from: https://www.sciencedirect.com/science/article/pii/S0025556417305473?via%3Dihub

30. Shcherbacheva A, Haario H. The Impact of Household Size on Malaria Reduction in Relation with Alterations in Mosquito Behavior by Malaria Parasite. J Multi-Valued Log Soft Comput. 2017;29:455–68.
