# Supplementary material for: Agent-based models of malaria transmission: a systematic review
Source: Malar J. 2018 Aug 17;17:299. doi: 10.1186/s12936-018-2442-y (PMC6098619; doi:10.1186/s12936-018-2442-y)
Supplement: Supplementary file 5 — Additional file 5. Overview of ensemble and comparative models. [file 12936_2018_2442_MOESM5_ESM.docx]

# Additional Information - Agent-Based Models of Malaria Transmission: A Systematic Review

Smith, Neal R.; Trauer, James M.; Gambhir, Manoj; Richards, Jack S.; Maude, Richard J.; Keith, Jonathan M.; Flegg, Jennifer A.

## Additional File 5: Overview of ensemble and comparative models.

| **Paper** | **Investigation** | **ABMs compared** | **# models** | **Ref.** |
| --- | --- | --- | --- | --- |
| Briet (2012) | Ensemble modelling | OpenMalaria | 14 | [1] |
| Smith(2012) | Ensemble modelling | OpenMalaria | 14 | [2] |
| Stuckey (2012) | Ensemble modelling | OpenMalaria | 14 | [3] |
| Briet (2013A) | Ensemble modelling | OpenMalaria | 14 | [4] |
| Briet (2013B) | Ensemble modelling | OpenMalaria | 14 | [5] |
| Briet (2013C) | Ensemble modelling | OpenMalaria | 14 | [6] |
| Stuckey (2014) | Ensemble modelling | OpenMalaria | 14 | [7] |
| Pemberton-Ross (2015) | Ensemble modelling | OpenMalaria | 6 | [8] |
| Penny (2015A) | Ensemble modelling | OpenMalaria | 6 | [9] |
| Yukich (2015) | Ensemble modelling | OpenMalaria | 14 | [10] |
| Cameron(2015) | Cross-comparison | OpenMalaria EMOD  Griffin *et. al* | 3 | [11] |
| Penny (2015B) | Ensemble modelling | OpenMalaria | 6 | [12] |
| Penny (2015C) | Cross-comparison | OpenMalaria  EMOD  Griffin *et. al*  Sauboin *et. al* | 4 | [13] |
| Stuckey (2016) | Ensemble modelling | OpenMalaria | 14 | [14] |
| Brady(2017) | Cross-comparison | OpenMalaria  EMOD  Griffin *et. al*  Maude *et. al* | 4 | [15] |

## References

1. Briët OJT, Hardy D, Smith TA. 2012 Importance of factors determining the effective lifetime of a mass, long-lasting, insecticidal net distribution: a sensitivity analysis. *Malar J* **11**(1):20. (doi:10.1186/1475-2875-11-20)

2. Smith T, Ross A, Maire N, Chitnis N, Studer A, Hardy D, Brooks A, Penny M, Tanner M. 2012 Ensemble modeling of the likely public health impact of a pre-erythrocytic malaria vaccine. *PLoS Med* **9**(1):e1001157.

3. Stuckey EM, Stevenson JC, Cooke MK, Owaga C, Marube E, Oando G, et al. 2012 Simulation of malaria epidemiology and control in the highlands of western Kenya. *Malar J* **11**(1):357–70. (doi:10.1186/1475-2875-11-357)

4. Briët OJT, Penny MA, Hardy D, Awolola TS, Bortel W Van, Corbel V, et al. 2013 Effects of pyrethroid resistance on the cost effectiveness of a mass distribution of long- lasting insecticidal nets : a modelling study. :1–12.

5. Briët OJT, Chitnis N. 2013 Effects of changing mosquito host searching behaviour on the cost effectiveness of a mass distribution of long-lasting , insecticidal nets : a modelling study. :1–11.

6. Briët OJT, Penny MA. 2013 Repeated mass distributions and continuous distribution of long-lasting insecticidal nets: modelling sustainability of health benefits from mosquito nets, depending on case management. *Malar J* **12**(1):401–19. (doi:10.1186/1475-2875-12-401)

7. Stuckey EM, Stevenson J, Galactionova K, Baidjoe AY, Bousema T, Odongo W, et al. 2014 Modeling the cost effectiveness of malaria control interventions in the highlands of western Kenya. *PLoS One* **9**(10). (doi:10.1371/journal.pone.0107700)

8. Pemberton-Ross P, Smith TA, Hodel EM, Kay K, Penny MA. 2015 Age-shifting in malaria incidence as a result of induced immunological deficit: a simulation study. *Malar J* **14**(1):287–300. (doi:10.1186/s12936-015-0805-1)

9. Penny MA, Galactionova K, Tarantino M, Tanner M, Smith TA. 2015 The public health impact of malaria vaccine RTS,S in malaria endemic Africa: country-specific predictions using 18 month follow-up Phase III data and simulation models. *BMC Med* **13**(1):170–89. (doi:10.1186/s12916-015-0408-2)

10. Yukich J, Chitnis N. 2015 When can malaria control and elimination programs safely reduce vector control efforts ? a simulation study . (July).

11. Cameron E, Battle KE, Bhatt S, Weiss DJ, Bisanzio D, Mappin B, et al. 2015 Defining the relationship between infection prevalence and clinical incidence of Plasmodium falciparum malaria. *Nat Commun* **6**:8170. (doi:10.1038/ncomms9170)

12. Penny MA, Pemberton-Ross P, Smith TA. 2015 The time-course of protection of the RTS,S vaccine against malaria infections and clinical disease. *Malar J* **14**(1):437. (doi:10.1186/s12936-015-0969-8)

13. Penny MA, Verity R, Bever CA, Sauboin C, Galactionova K, Flasche S, et al. 2016 Public health impact and cost-effectiveness of the RTS,S/AS01 malaria vaccine: A systematic comparison of predictions from four mathematical models. *Lancet* **387**(10016):367–75. (doi:10.1016/S0140-6736(15)00725-4)

14. Stuckey EM, Miller JM, Littrell M, Chitnis N, Steketee R. 2016 Operational strategies of anti-malarial drug campaigns for malaria elimination in Zambia’s southern province: a simulation study. *Malar J* **15**(1):148–61. (doi:10.1186/s12936-016-1202-0)

15. Brady OJ, Slater HC, Pemberton-Ross P, Wenger E, Maude RJ, Ghani AC, et al. 2017 Role of mass drug administration in elimination of Plasmodium falciparum malaria: a consensus modelling study. *Lancet Glob Heal* **5**(7):e680–7. (doi:10.1016/S2214-109X(17)30220-6)
